# Emerin Is Required for Proper Nucleus Reassembly after Mitosis: Implications for New Pathogenetic Mechanisms for Laminopathies Detected in EDMD1 Patients

**DOI:** 10.3390/cells8030240

**Published:** 2019-03-13

**Authors:** Magda Dubińska-Magiera, Katarzyna Kozioł, Magdalena Machowska, Katarzyna Piekarowicz, Daria Filipczak, Ryszard Rzepecki

**Affiliations:** 1 Laboratory of Nuclear Proteins, Faculty of Biotechnology, University of Wroclaw, Fryderyka Joliot-Curie 14a, 50-383 Wroclaw, Poland; magda.dubinska-magiera@uwr.edu.pl (M.D.-M.); katarzyna.koziol@uwr.edu.pl (K.K.); magdalena.machowska@uwr.edu.pl (M.M.); katarzyna.piekarowicz@uwr.edu.pl (K.P.); dariafilipczak2@gmail.com (D.F.); 2 Department of Animal Developmental Biology, Institute of Experimental Biology, University of Wroclaw, Sienkiewicza 21, 50-335 Wroclaw, Poland

**Keywords:** emerin, EDMD1, lamin, laminopathy, lamin A/C, LAP2β, tubulin, mitotic spindle

## Abstract

Emerin is an essential LEM (LAP2, Emerin, MAN1) domain protein in metazoans and an integral membrane protein associated with inner and outer nuclear membranes. Mutations in the human *EMD* gene coding for emerin result in the rare genetic disorder: Emery–Dreifuss muscular dystrophy type 1 (EDMD1). This disease belongs to a broader group called laminopathies—a heterogeneous group of rare genetic disorders affecting tissues of mesodermal origin. EDMD1 phenotype is characterized by progressive muscle wasting, contractures of the elbow and Achilles tendons, and cardiac conduction defects. Emerin is involved in many cellular and intranuclear processes through interactions with several partners: lamins; barrier-to-autointegration factor (BAF), β-catenin, actin, and tubulin. Our study demonstrates the presence of the emerin fraction which associates with mitotic spindle microtubules and centrosomes during mitosis and colocalizes during early mitosis with lamin A/C, BAF, and membranes at the mitotic spindle. Transfection studies with cells expressing EGFP-emerin protein demonstrate that the emerin fusion protein fraction also localizes to centrosomes and mitotic spindle microtubules during mitosis. Transient expression of emerin deletion mutants revealed that the resulting phenotypes vary and are mutant dependent. The most frequent phenotypes include aberrant nuclear shape, tubulin network mislocalization, aberrant mitosis, and mislocalization of centrosomes. Emerin deletion mutants demonstrated different chromatin binding capacities in an in vitro nuclear assembly assay and chromatin-binding properties correlated with the strength of phenotypic alteration in transfected cells. Aberrant tubulin staining and microtubule network phenotype appearance depended on the presence of the tubulin binding region in the expressed deletion mutants. We believe that the association with tubulin might help to “deliver” emerin and associated membranes to decondensing chromatin. Preliminary analyses of cells from Polish patients with EDMD1 revealed that for several mutations thought to be null for emerin protein, a truncated emerin protein was present. We infer that the EDMD1 phenotype may be strengthened by the toxicity of truncated emerin expressed in patients with certain nonsense mutations in *EMD*.

## 1. Introduction

Nuclear lamina and nuclear membrane proteins interacting with lamins play important roles in most cellular and intra-nuclear processes, including spatial organization of chromatin, regulation of DNA replication and transcription, RNA splicing, and nuclear transport [1]. Lamins and lamina-associated proteins are also involved in intracellular signaling and regulate metazoan development (reviewed in [2,3]). Their function in the inner nuclear membrane (INM) during nuclear disassembly and reassembly in mitosis is less well known [4,5,6,7,8,9,10]. Nevertheless, the role of lamins in nuclear disassembly and reassembly is much better understood than the role of INM proteins interacting with lamins [8,11,12,13,14,15,16].

One of the biggest groups of lamin-interacting proteins comprises the integral membrane proteins of the nuclear envelope (NE) and nuclear lamina (NL). The best known of these are the LEM domain proteins (LAP2, Emerin, MAN1) [17,18], which are evolutionarily conserved and present in yeasts, plants, and all metazoans. LAP2 isoforms and MAN1 (LEMD) isoforms are fully conserved while emerin is not [19,20].

Emerin is one of the key components of the nuclear lamina. Mutations in the human *EMD* gene coding for emerin result in the genetic disorder Emery–Dreifuss muscular dystrophy type 1 (EDMD1, OMIM 310300) [21,22,23,24]. This rare disease belongs to a broader group called laminopathies—a heterogeneous group of rare genetic disorders with over 11 distinct phenotypes affecting tissues of mesodermal origin, of which the most severe are thought to be restrictive dermopathy, Hutchison–Gilford progeria syndrome (HGPS) and progeroid laminopathies [25].

EDMD1 is a rare, degenerative myopathy characterized by muscle weakness and atrophy, early joint contractures, and usually cardiac involvement (conduction block) but with no nervous system defects. EDMD1 is X-linked, and most identified mutations are frameshift, nonsense, or splice site [26]. In most cases, emerin is undetectable by immunostaining in muscle biopsies [27,28]. In the case of mouse models of EDMD1, representing the null phenotype for emerin, only minor symptoms are detected, and affected mice are almost indistinguishable from controls [29,30]. The protein Lmo7, which is expressed in mouse, might possibly offer compensation of emerin loss in these models [31]. Regardless, this discrepancy between the mouse model of EDMD1 and the human phenotype suggests other disease mechanisms, potentially involving missense and nonsense mutations, rather than the total loss of function of emerin or emerin protein loss. Other genetic factors, together with short lifespan, may also be crucial for generating the disease phenotype in mice.

**Figure 1:**
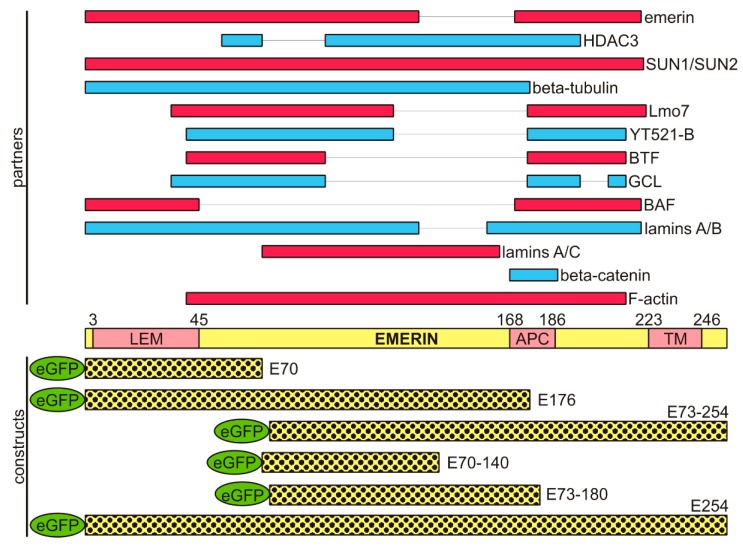
Functional domains identified in emerin, emerin domains identified as necessary for interaction with other nuclear proteins, and constructs used in this study. Emerin contains a LEM domain [32,33] on its very N-terminus, followed by a so-called LEM-like domain located within the functional lamin-binding domain. The Adenomatous Polyposis Coli (APC)-like domain, responsible for interaction with β-catenin, localizes to fragment 168–186 aa residues, and the transmembrane domain localizes to 223–246 aa residues. Upper: emerin interactions and mapped emerin domains necessary for the interactions. Lower: the set of genetic constructs prepared in our laboratory and used for the study. LEM—LAP2 Emerin MAN1 domain; APC—domain necessary for interaction with β-catenin and Wnt signaling; TM—transmembrane domain; EGFP—the position of the EGFP protein fused to emerin proteins. Numbering represents amino acid residue numbers present in a particular construct. E70—deletion mutant containing amino acid residues from 1 to 70; E70–140—a construct containing amino acid residues from 70 to 140. The rest of the mutants are designated following the same pattern.

Emerin is an integral membrane protein localized during interphase to the inner and outer nuclear envelopes. Schematic diagrams of the functional domains identified in the emerin and of emerin fragments identified as responsible for interactions with other proteins are shown in Figure 1.

Emerin is involved in several processes through interactions with many partners [34,35] (Figure 1). It interacts with BAF through the LEM domain [36,37,38] and with lamins through the mapped lamin-binding domain [39]. Emerin interacts with BAF and chromatin as a dimer [40] and is thought to interact with many other proteins including LUMA [41], HDAC3 (histone deacetylase 3) [42], Btf [43], GCL [44], actin [45], Lmo7 [46], NET25 [47], and β-catenin [48] (see also [35]). The recently resolved structure of the ternary complex of BAF, LEM domain of emerin, and lamin A [40] suggests that the same complexes may exist and function also in vivo.

Studies in *Caenorhabditis elegans* have shown that lamin, LEM domain proteins, and BAF are required for one another’s activity during nuclear assembly following metaphase [49,50,51]. Over the last decade, the focus has been limited to the possible involvement of nuclear lamina and envelope proteins in the regulation and spatial organization of mitotic spindle assembly and function and nuclear envelope disassembly and reassembly. Yet this involvement could be crucial to a better understanding of the disease process underlying EDMD1.

Our collaborative studies using in vitro nuclear assembly of the *Xenopus* oocyte system have demonstrated that during mitosis, different fractions of membrane vesicles vary in protein composition [52]. Previous studies using the same system showed that deletion mutants of human and *Xenopus* LAP2 protein inhibit in vitro nucleus reassembly [53,54,55,56,57,58]. Similar studies with emerin deletion mutants have also shown that emerin deletion mutants inhibit nuclear reassembly in vitro and chromatin decondensation and inhibit NPC reassembly [52]. The strongest inhibitory effect was found for the E1–70 emerin fragment (LEM domain). No visible effects on nucleus reassembly in an in vitro system were observed for deletion mutant E73–180 (lamin/tubulin/actin binding region).

Location of emerin in cells and cell nuclei was initially studied using fluorescence microscopy, demonstrating the location of the protein in the nuclear envelope (NE) during interphase and at various locations during metaphase with possible colocalization with tubulin staining, but it is hard to interpret the presented data convincingly [59]. The study by Haraguchi and coworkers [60] presented data on anaphase and telophase location of GFP-emerin fusion protein on decondensing chromatin using living cells. Another live fluorescent study by the same group demonstrated that selected deletion mutants of emerin as fusion proteins with GFP can associate with decondensing chromatin and “core” regions of chromatin except full length emerin lacking the LEM domain which showed uniform staining at chromosomes [11]. The same group conducted time lapse live fluorescent microscopy of all major nuclear proteins as fusion proteins with GFP from early anaphase to late telophase [61], demonstrating, with the use of transmission electron microscopy (TEM), that BAF, microtubules and membranes can be detected at the same loci at decondensing chromatin. They also reported the colocalization of the fraction of BAF-GFP with spindle microtubules at metaphase and telophase in fixed cells but not during live imaging [61]. Another study by Salpingidou and coworkers demonstrated the role of emerin in centrosome anchorage at the NE through interactions with tubulin at the outer nuclear membrane (ONM) [62]. The tubulin interacting domain in emerin was mapped to the emerin 1–176 fragment (E176 deletion mutant) encompassing the LEM domain and the entire lamin A/C binding domain (Figure 1).

Therefore, we focused our study on thorough analyses of the interplay between emerin, BAF, tubulin, and membranes during mitosis and the effect of the presence of emerin deletion mutant on nucleus structure, the tubulin network, and centrosomes location.

To address these questions, we used a tissue culture model system to assess the location of emerin and lamins during mitosis and NE reassembly and determine whether dysfunctional emerin protein (emerin deletion mutants) affects nuclear structure and function.

Here we show that emerin, together with BAF and membranes, colocalize with mitotic spindle microtubules, and functional emerin is required for proper nuclear organization and that its loss leads to increased frequency of aberrant cellular phenotypes. As expected, the expression of E70 deletion mutant had a dominant-negative effect on cell phenotype, but E176 and E70–140 mutants had a surprisingly similar effect. The E73–254 mutant also influenced cellular phenotype, but more weakly. Results of an in vitro chromatin binding assay based on *Xenopus* chromatin and extracts revealed that no emerin deletion mutant tested except E220 (nucleoplasmic full length) could bind to chromatin without its prior modification by *Xenopus* extract. In addition, emerin, along with other proteins of INM and lamins, transiently associates with mitotic spindle microtubules and related membranes. We also show that mitotic location of emerin, BAF, and lamins is protein specific and unique for the particular protein and does not fully follow the distribution of the soluble protein fraction or bulk membranes during mitosis. Our preliminary data for cells from a Polish cohort of patients with EDMD1 suggest the presence of truncated emerin proteins with at least some of the so-called emerin-null mutations.

## 2. Materials and Methods

### 2.1. Plasmids

We investigated the following pEGFP-C1 constructs with cloned fragments of the human emerin gene (*EMD*): pEGFP-emerin 1–254 (E1–254, full-length emerin), pEGFP-emerin 1–70 (E70), and pEGFP-emerin 1–176 (E176), all kindly shared by J. A. Ellis [63]; and pEGFP-emerin 70–140 (E70–140), pEGFP-emerin 73–180 (E73–180), and pEGFP-emerin 73–254 (E73–254), all cloned in our laboratory [52].

### 2.2. Cell Culture and Transfection

HeLa cells were grown in MEMα medium containing 10% fetal bovine serum (FBS) (Sigma, Saint Louis, MO, USA), 1% GlutaMAX (Gibco, Waltham, MA, USA), 100 U/mL penicillin, 100 μg/mL streptomycin, and 0.25 μg/mL amphotericin B (Gibco) at 37 °C, 5% CO_2_, and under humid conditions. Transfection of HeLa cells was carried out with Metafectene Pro (Biontex, Munich, Germany) or Turbofect (Thermo Scientific, Waltham, MA, USA) using a 1 μg:6 μL or 1 µg:2 µL ratio of plasmid DNA:transfection reagent respectively. Cells were grown on 6-well plates at an initial density of 6 × 10^4^ per well and processed after 24, 48, 72, or 96 h after transfection, depending on the experiment.

Human dermal fibroblasts were derived from chest skin biopsies of EDMD1 patients, obtained during pacemaker replacement. Normal human dermal fibroblasts (NHDF) were purchased from Lonza. Fibroblasts were obtained and cultivated under the same conditions as HeLa cells. Patient cells were collected according to ethical and medical standards set by Polish law and based on approval of the ethical committee (DOP-GMO.431.85.2018; DOP-GMO.431.79.2018) and after obtaining written consent from the patients.

### 2.3. Immunofluorescence

For immunofluorescence (IF), HeLa cells were grown on 12-mm coverslips at an initial density of 2.5 × 10^5^ per well on 6-well plates. At 48 h after transfection, cells were fixed with 4% PFA in phosphate-buffered saline (PBS) for 15 min at room temperature (RT), permeabilized with 0.5% Triton X-100 in PBS (*v*/*v*) for 5 min at 4 °C, and blocked with 1% FBS in PBS (for 30 min at RT). All wash steps were done with PBS.

Primary antibodies were used for overnight incubation at 4 °C and secondary antibodies for 60 min at RT. DNA was stained with DAPI (4,6-diamidino-2-phenylindole, 0.2 μg/mL in mounting medium with Mowiol and DABCO). For imaging, the confocal microscopes LSM 510 META with the FCS system and Olympus FluoView FV1000 were used. Any brightness and contrast adjustments were performed in Adobe Photoshop, Zen 2007 (Carl Zeiss, Oberkochen, Germany) or ImageJ [64].

The fibroblasts were seeded on 12-mm coverslips to obtain 50% confluence, then fixed after 24 h and stained as described for HeLa cells. For imaging, the confocal microscope Leica SP8 (Leica Camera AG, Wetzlar, Germany) with LasX software was used. Z-stacks were presented as maximum intensity projection images.

### 2.4. SDS-PAGE and Western Blotting

Cells were collected by trypsinization, counted, washed in PBS, and centrifuged at 500× *g* for 5 min. The cell pellet was resuspended in Laemmli buffer and incubated for 10 min at 96 °C. Electrophoretic separation of proteins was carried out on a polyacrylamide gel (12% separating gel, 6% stacking gel) under denaturing conditions (SDS-PAGE), according to the Laemmli method, using Tris-Glycine-SDS (TGS) buffer.

A wet electrotransfer was performed in a cold TGS buffer with 20% methanol, using a PROTRAN nitrocellulose membrane with a pore size of 0.2 μm. Membranes were blocked in 5% non-fat milk in a PBST (PBS with 1% Tween 20) and incubated in a primary antibody solution in PBST, 5% bovine serum albumin in PBST, or 5% non-fat milk in PBST overnight at 4 °C; secondary antibodies diluted in 5% non-fat milk in PBST were applied for 1 h at RT. Visualization was carried out using luminescence; the membrane was incubated in a Clarity Western ECL substrate for 5 min, and then the signal was detected using the ChemiDoc MP imaging system.

### 2.5. Antibodies

For IF, the following antibodies were used: rabbit anti-E70 (anti-1–70 aa of human emerin, 1:400) made in our laboratory, rabbit anti-emerin 66–252 (1:40, Proteintech, Manchester, UK), rabbit anti-emerin N-terminal (1:400, Cell Signaling), mouse anti-β-tubulin (1:150, Sigma T4026), rabbit anti-α-tubulin (4a 1:300, Invitrogen PA5-29444, Carlsbad, CA, USA), rabbit anti-β-tubulin (1:300, Invitrogen), rabbit anti-BAF (R449, 1:30, [65], a kind gift of P.A. Fisher, SUNY SB (Stony Brook, NY USA)), mouse anti-lamin A (1:10, Novus Biologicals LLC (Centennial, CO, USA), mouse anti-lamin A/C (Jol2, 1:25, kind gift of prof. C.J. Hutchison, Durham, UK [66]), rabbit anti-pericentrin (1:500, Abcam, Cambridge, UK), mouse anti-TPX2 (1:200, Santa Cruz Biotechnology, Dallas, TX, USA), goat anti-lamin B1 (1:50, Santa Cruz Biotechnology), mouse anti-emerin (anti-nucleoplasmic domain, 1:50, NCL Novocastra, Newcastle Upon Tyne, UK), and mouse anti-LAP2 (LAP12, 1:20, [67,68]).

For actin staining, phalloidin conjugated with rhodamine was used. Cellular lipid membranes were stained with DHCC (3,3′’-dihexyloxacarbocyanine iodide at a concentration of 7.5 μg/mL; Invitrogen).

For immunoblotting, the following antibodies were used: rabbit anti-emerin 66-252 (1:1000, Proteintech), rabbit anti-emerin N-terminal (1:5000, Cell Signaling), mouse anti-α actin (1:400, Sigma-Aldrich, Saint Louis, MO, USA), and rabbit anti-α-tubulin (4a, 1:5000, Invitrogen). Affinity-purified antibodies anti-E70 and E220 (1:1000) were generated in rabbits against bacterially expressed emerin 1–70 fragment and emerin 1–220 fragment. Affinity purified rabbit antibodies were generated for the XLAP2 “common domain” in our laboratory [58].

Secondary antibodies for IF and immunoblotting were as follows: donkey anti-rabbit Alexa Fluor 488 and donkey anti-mouse Alexa Fluor 488 (both 1:100, Jackson ImmunoResearch, West Grove, PA, USA), donkey anti-mouse and donkey anti-rabbit TRITC (both 1:50, Jackson ImmunoResearch), donkey anti-mouse and donkey anti-rabbit DyLight 647 (both 1:50, Jackson ImmunoResearch), donkey anti-mouse HRP (1:3000, Santa Cruz Biotechnology), and goat anti-rabbit HRP (1:15,000, Jackson ImmunoResearch).

### 2.6. Laser Scanning Confocal Microscopy Analyses

Most of the immunofluorescence imaging was performed using a Zeiss LSM510 Meta confocal microscope equipped with four lasers: 405 nm (diode), 488 nm (Argon), 561 nm (DPSS) 561-10 and 633 nm (HeNe). Some of the imaging was also performed using an Olympus FluoView FV1000 confocal laser scanning microscope equipped with four lasers: 405 nm (diode), 473 nm (diode), 559 nm (diode), 635 nm (diode). The images were recorded by employing a 60× oil-immersion objective (Olympus Plan-Apochromat UPLSAPO 60X/1.35/0,17/FN 26,5). For imaging on the Zeiss LSM510 Meta we used a 63× objective (Zeiss Plan Apochromat 63×/1.40; Oil Dic M27). We did not use the Meta detector. Typically, we used frame scan mode, collecting each channel separately with pixel dwell time between 12.8 and up to 51.2 µs at resolution 1024 for 1024 and 8 bit depth. Airy units were set for each channel to obtain always the desired optical thickness (typically 1 µm thick section; 1.5 µm section for entire spindle visualization). In order to avoid any potential bleed-through we scanned each channel separately. For Leica SP8 confocal microscope we used also four lasers: 405 nm (diode), 488 nm (Argon), 561 nm (DPSS) and 633 nm (HeNe). For imaging we used 63× objective (HC PL APO CS2 63×/1.40 OIL). Typically, we used frame scan mode, collecting each channel separately with pixel dwell time around 12 µs at resolution 1024 for 1024 and bit depth 8bit. Additionally, for visualization of proteins we used secondary antibodies with fluorochromes emitting in different wavelengths, which allowed us to visualize emerin using different combinations of channels. In all documentation we always use the same color codes for particular channels: blue for DAPI/405 nm-excitation channel, green for argon/488 nm-excitation channel, yellow for 561 nm-excitation channel and red for 633 nm-excitation channel in 4-channel mode. In 3-channel mode the 561 nm excitation channel was coded in red.

### 2.7. In Vitro Nuclear Assembly and Chromatin Binding Assay

Protein expression and purification from a bacterial system were performed as described previously [57,69]. In vitro nuclear assembly reactions and the in vitro chromatin binding assays were performed as described previously [52,57,58].

### 2.8. Phenotype Analysis

The phenotypic evaluation was performed on HeLa cells, in which, based on the GFP signal, overexpression of emerin variants was found. Observers blinded to the plasmids used in the transfection experiment assessed cell phenotypes. The phenotypic analysis included assessment of cell shape, cytoskeleton, centrosome locations, and morphology of the cell nucleus and nuclear envelope. Phenotypes of transfected cells differing from phenotypes of untransfected cells with respect to the above criteria were considered “improper.” The assessment was repeated three times for each cell, with n = 20 cells per plasmid.

### 2.9. Phenotype Quantitative and Statistical Analysis

For statistical analysis, HeLa cells were transfected, stained, and visualized as described. Based on observations, three main incorrect phenotypes were distinguished: “abnormal” with an improperly shaped nucleus, distinctive often also for redistribution of nuclear envelope proteins (lamins and emerin) from one pole of the nucleus; “cut-like,” with cells that have not totally separated after mitosis; and the apoptotic-shaped cells. See also [58].

Phenotypes were counted for each investigated emerin deletion mutant (E70, E176, and E70–140), cells with overexpressed recombinant E1–254 (full-length emerin), and normal (untransfected) HeLa cells. On average, 350 transfected cells and 600 untransfected cells were taken into account. Such estimations were conducted based on three independent experiments. Based on counting results, to assess the significance of differences between numbers of untransfected, E254, and emerin deletion mutant phenotypes the two-tailed Student’s *t* test was used, *p* < 0.05.

### 2.10. Cell Cycle Analysis

HeLa cells were transfected according to the described procedure on 6-well plates. At 48 h after -transfection, cells were collected by trypsinization and centrifuged (5 min, 4 °C, 1000 rpm). The pellet was suspended in PBS buffer and ice-cold methanol added dropwise in a 1:5 ratio. Fixation was carried at −20 °C for 30 min. After addition of two volumes of PBS to the suspension, cells were spun down as previously described. The second washing step was with 5 mL of PBS under the same centrifugation conditions.

Fixed cells were stained by addition of propidium iodide (PI) to 25 µg/mL and RNase A (to 100 µg/mL) to the final cell suspension and incubated for 5 min on ice. Then the last two washing steps were done with 5 mL of PBS each. The obtained pellet was suspended in 1 mL of PBS and analyzed using the flow cytometer Coulter Epics XL-MCL (Beckman Coulter Inc., Brea, CA, USA). The experiment was repeated three times independently.

For each cell cycle phase, GFP-positive and GFP-negative cells were gated and counted. Based on these data, the cell cycle distribution for transfected and untransfected cells, separately, was obtained. In the subsequent step, statistical analysis of the significance of differences between untransfected, E254, and emerin deletion mutant populations of cells were performed using Student’s *t*-test, *p* < 0.05 or *p* < 0.005.

## 3. Results

### 3.1. Emerin and Interacting Protein BAF Transiently Associate with Mitotic Spindle Microtubules, Centrosomes, and Associated Membranes during Mitosis

**Figure 2:**
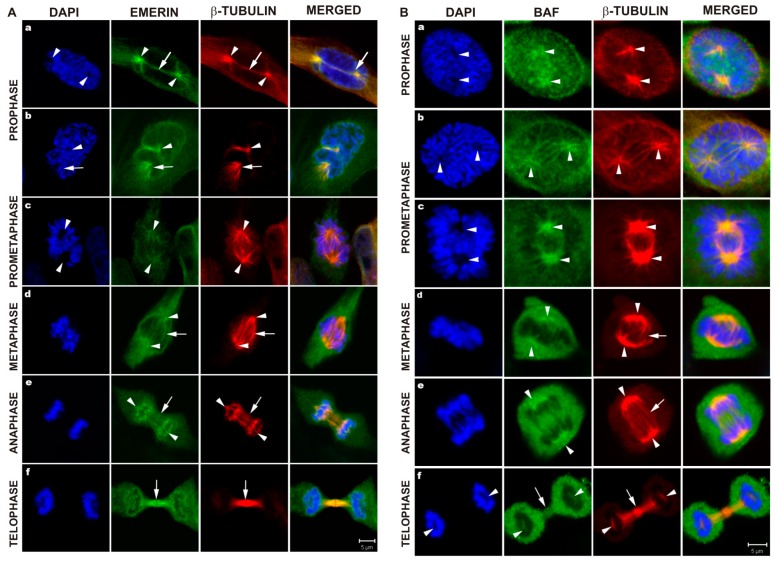
Cell cycle-dependent distribution of emerin and BAF in HeLa cells. (**A**) A fraction of emerin colocalizes with centrosomes and spindle microtubules during mitosis. Cells representing typical emerin phenotypes for particular mitosis stage were taken in order to demonstrate a fraction of emerin associated with mitotic spindle and centrosomes. Arrowheads indicate the position of centrosomes (a–e), arrows indicate the spindle microtubule (a,b,d,e) and midbody (f). (**B**) Emerin binding partner BAF colocalizes with centrosomes and spindle microtubules. Arrowheads indicate the position of centrosomes (a–f), arrows indicate spindle microtubule (d,e) and midbody (f). Note the change in staining character for BAF from “grainy” (a–c) to “smooth” (d–f). Note the colocalization between polar microtubule and emerin very clearly visible between centrosomes (A, a, arrow) and nuclear envelope invagination (A, b, arrow) associated with centrosomes and astral microtubules in NE invaginations. Arrows point to microtubules associated with emerin. HeLa cells were stained with antibodies against emerin (green, A), BAF (green, B), and β-tubulin (red). Single Z-stacks, 1.5 μm through cell nucleus or mitotic spindle centered at the centrosomes were visualized. Scale bar, 5 μm.

Because previous reports, including ours, demonstrated an association of emerin with tubulin (see Introduction), we specifically focused our analyses using laser multichannel confocal microscopy on the distribution of emerin and tubulin during mitosis as the least studied part of the cell cycle with respect to emerin location. During mitosis, the fraction of emerin colocalized with centrosomes (Figure 2A, a–d, arrowheads) entering the nuclear space at prophase, and with spindle microtubules associated with the mitotic spindle (Figure 2A, a–e, arrows). Emerin also colocalized with centrosomes and spindle microtubules during prometaphase (Figure 2A, c) and metaphase (Figure 2A, d). At anaphase, a fraction of emerin still associated with centrosomes (Figure 2A, e, arrowheads), but major fractions of visible emerin associated with spindle microtubules attached to decondensing chromatin and chromatin “core regions” next to the centrosomes, and chromatin in the region connected to spindle microtubules. Note the colocalization of emerin with microtubules at the mitotic spindle between separating chromatids and especially at the *foci* on decondensing chromatin where microtubules are connected to chromatin in so-called “core regions” with a high level of bound emerin while the non-“core region” showed very weak or no staining for emerin (Figure 2A, e). See also Supplemental Figure S1 for demonstrated colocalization of emerin, spindle microtubules and membranes.

Because BAF interaction with emerin through the LEM domain is essential for nuclear envelope breakdown (NEBD) and reassembly, and there is a report about the colocalization of BAF-GFP protein with the mitotic spindle during metaphase and anaphase [61], we analyzed the mitotic distribution of BAF together with β-tubulin. At prophase, nuclear BAF localized in the NE and chromatin, dispersed inside the nucleus, with increased density in centrosomal and mitotic spindle regions (arrowheads) (Figure 2B, a). At prometaphase, a fraction of BAF colocalized with centrosomes and spindle microtubules, forming “aster”-like structures (Figure 2B, b) and gradually lost its association with the NE (compare Figure 2B, b with Figure 2B, c). At metaphase and anaphase, a major fraction of BAF was dispersed within the cytoplasm, and a minor fraction colocalized with centrosomes and chromatin next to centrosomes (Figure 2B, d,e; arrowheads). At telophase, BAF was predominantly dispersed in the cytoplasm (Figure 2B, f), with a fraction of the protein colocalized with tubulin at decondensing chromatin and spaces in the decondensing chromatin emptied by centrosomes with traces of tubulin still visible.

The presence of emerin at mitotic spindle microtubules and invagination sites associated with centrosomes (and microtubules) entering the nuclear space immediately raises the question of whether NE membranes are also associated with emerin at these locations. To address this question, we analyzed the mitotic distribution of membranes, stained with the lipophilic dye DHCC, together with emerin and the mitotic spindle. For mitotic spindle visualization, for technical reasons, we used antibodies for TPX2 protein, which associates with spindle microtubules at late prophase/early prometaphase (Figure S1). The level of TPX2 at the spindle is lower than that of tubulin, and TPX2 does not interact with emerin according to our data (unpublished conference report; Koziol K. et al.). At prophase (Figure S1b), we detected one very well-formed site of NE invagination; the other one is barely visible, (arrowheads), associated with the entering centrosome, associated with membranes that colocalize with emerin. The second invagination is being formed already to the left (second arrowhead). The bulk emerin fraction is still associated with the NE although a small fraction of emerin is already dispersed within cytoplasm compared to interphase staining. At later stages, a fraction of membranes was associated with emerin at the mitotic spindle and centrosome locations (Figure S1c,d prometaphase, metaphase) and later at anaphase and telophase (Figure S1e,f), and at decondensing chromosome surfaces in “core” regions, where emerin and membranes colocalized with spindle microtubules. “Non-core” regions of decondensing chromatin surface were not associated with emerin, membranes, or tubulin at anaphase (Figure S1).

### 3.2. Lamin A/C Transiently Associates with Emerin and Mitotic Spindle Microtubules and Membranes

**Figure 3:**
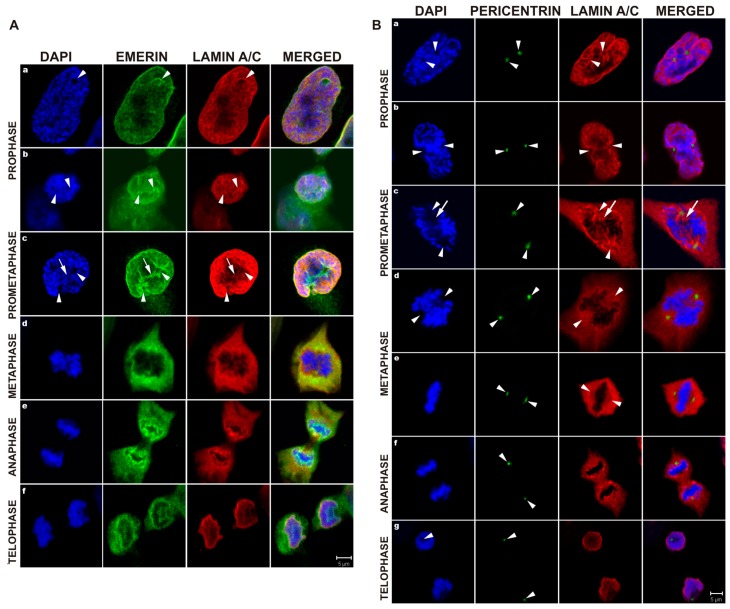
Cell cycle-dependent distribution of emerin and pericentrin in comparison to lamin A/C in HeLa cells. (**A**) Emerin colocalizes with lamin A/C during mitosis. Single confocal sections centered at centrosomes located at the nuclear envelope at prophase and prometaphase are shown in order to demonstrate the distribution of emerin and lamin next to centrosomes at the nuclear envelope. At metaphase to anaphase the confocal section was centered at the mitotic spindle. Arrowheads indicate the nuclear envelope invagination formed by moving centrosomes into the nuclear space (a–c), and arrow indicates the nuclear envelope fragment and nucleus section containing emerin but free of polymerized lamin A/C associated with forming the mitotic spindle between centrosomes inside the cell nucleus (c). At prophase and prometaphase, emerin colocalizes with lamin A/C in the regions of the nuclear envelope and associated with NE centrosomes. At prometaphase, when centrosomes have already migrated inside the cell nucleus, membranes and emerin are still associated with centrosomes and mitotic spindle microtubules entering the nuclear space (prometaphase, c) while lamin A/C colocalization is gradually lost (arrow on c). These regions showed a weaker signal for lamin A/C (possibly because of depolymerization and diffusion of lamins) while the emerin signal remained at a steady level. The bulk of the protein fraction was dispersed all over the cell until anaphase (d–e), when proteins started to associate with decondensing chromatin. At telophase, all lamin A/C proteins were associated with or around chromatin, while a fraction of emerin still remained cytoplasmic (f). (**B**) Distribution of lamin A/C in relation to centrosomes during mitosis in HeLa cells. Pericentrin (arrowheads) indicates the position of centrosomes during mitotic division (a–f). Single confocal sections centered at centrosomes were shown throughout mitosis. Centrosome positions are visualized by arrowheads. Arrow demonstrate the position of remnants of the fragmented nuclear lamina structure associated with spindle microtubules at one spindle pole (c). Note the nuclear lamina invaginations associated with centrosomes entering the nuclear space at prophase and not fully depolymerized nuclear lamina still surrounding the cell nucleus at prometaphase, which confirms previous reports on the association of nuclear lamina and emerin with membranes with microtubules at prophase. HeLa cells were stained with antibodies against emerin (green, A), pericentrin (green, B), and lamin A/C (red). Single section, 1.5 μm, were taken centered at spindle microtubules or centrosomes. Scale bar, 5 μm.

Emerin interacts with lamins A and C, that are major components of nuclear lamina, so we decided to address the question of the fate of NL structure connected to NE membranes and emerin. Figure 3A demonstrates the laser scanning confocal microscopy visualization of location of lamin A/C and emerin in relation to centrosomes (arrowheads) entering the nuclear space.

At prophase and prometaphase, emerin colocalizes with lamin A/C in the regions of centrosomes and mitotic spindle microtubules (astral microtubules) entering the nuclear space (prometaphase). Note two clearly well visible invaginations (Figure 3A, c, arrowheads) with a connecting furrow between them, very well stained for emerin, while lamin A/C structures at these locations are almost fully dispersed and weakly stained (Figure 3A, c, arrow). This might be possible because of depolymerization and diffusion of lamins in these regions while the emerin signal remained at a steady level (Figure 3A, c). The bulk of the protein fraction of lamin A/C and emerin was dispersed in the cytoplasm from metaphase until anaphase, when proteins started to associate with decondensing chromatin (Figure 3A, d,e). At telophase, all lamin A/C proteins were associated with or around chromatin, while a fraction of emerin still remained cytoplasmic (Figure 3A, f).

In order to visualize better lamins A/C as a marker of the NL in relation to centrosome location, we stained mitotic cells for pericentrin (Figure 3B). NL structures were associated with entering centrosomes, and formation of aster-like structures by the NL was visualized by staining for lamin A/C (Figure 3B, a,b, arrowheads). The staining pattern resembles the membrane/lamina structures dispersed on microtubules demonstrated earlier by Haraguchi and coworkers [61]. At prometaphase, NL fragments (or sheets) entered the nuclear space together with centrosomes and associated aster microtubules (Figure 3B, c, arrows). At early metaphase, most of the NL structure was dispersed, but a fraction of lamin A/C remained at the outside surface of chromosomes (Figure 3B, d) and another associated with the centrosomal region of the mitotic spindle (Figure 3B, e, metaphase). At anaphase, reassociation of lamins A/C and the NL started at the chromatin and continued at telophase (Figure 3B, f,g).

Taken together, the results indicate that emerin, BAF, and lamins A/C transiently associate with mitotic spindle microtubules, centrosomes, and associated membranes during mitosis.

### 3.3. Emerin, Unlike another LEM Domain Protein, LAP2β, Follows the Mitotic Spindle and Not the Membrane

Previous reports demonstrated metaphase location of BAF-GFP protein in the mitotic spindle [61] while our current study demonstrates the endogenous BAF fraction associated with the mitotic spindle. The published data obtained during initial studies of emerin during mitosis might suggest mitotic colocalization of emerin with microtubules [59].

Since emerin was found to interact with tubulin [62] and we report here also colocalization of emerin with mitotic spindle tubulin and membranes, it was of interest whether emerin partner BAF may recruit other LEM domain proteins to the mitotic spindle.

To test this, we performed comparative analyses of the colocalization of emerin and LAP2β (another nuclear integral membrane protein containing a LEM domain) with membranes during mitosis. Figure S2 illustrates the mitotic distribution of the membranes and both proteins.

During interphase, both proteins localized to the NE/NL fraction, which was also stained by DHCC (Figure S2a). A small fraction of emerin and LAP2β was also found in the cytoplasm. At prophase (Figure S2b), a small fraction of emerin and LAP2β showed divergent localization, with the latter perfectly following the membrane distribution (including inside the nuclear space). Arrowheads point to other typical locations inside the nucleus where LAP2β colocalizes with membrane fractions. Emerin is either not present in these membranes or shows very weak staining. Arrows point to the expected location of centrosomes of which the upper one is also associated with membranes and contains emerin. The other emerin loci colocalize with the other centrosome but are absent from membranes. Staining at the NE/NL was well preserved. At prometaphase (Figure S2c), LAP2β and membranes staining represent a similarly diffused pattern through cytoplasm and spaces not occupied by chromosomes while the emerin fraction despite the diffuse pattern was also associated with two distinct foci associated with centrosomes (Figure S2c, arrows).

During metaphase (Figure S2d), the location of membranes, LAP2β and emerin is similarly diffused with a fraction associated with the mitotic spindle at the center of the cell (arrows).

During anaphase and telophase, LAP2β and emerin gradually assemble at the decondensing chromatin with membranes also associated with them (Figure S2e,f). Interestingly, diffused, cytoplasmic staining for emerin is much stronger than for LAP2β. Proportionally, the LAP2β staining at the chromatin foci (arrowheads) is more intense than for emerin. See also staining for emerin, but not LAP2β in the midbody at telophase (Figure S2f).

A similar distribution of LAP2 proteins and BAF to that observed by us was reported previously [67,70]. The only exception was the specific location of emerin in the midbody, a structure rich in polymerized tubulin, where only emerin (not LAP2) was present. Therefore, this finding confirms the preferential interaction of tubulin with emerin over LAP2β.

### 3.4. EGFP-Emerin Transfection Studies Confirm Antibodies-Based Observations

**Figure 4:**
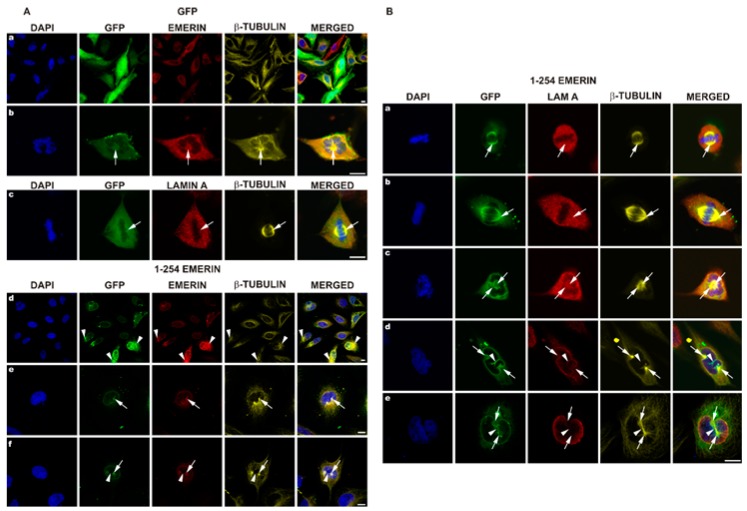
Transfected emerin-EGFP fusion protein associates with microtubules, centrosomes, and mitotic spindle microtubules. HeLa cells were transfected with plasmids encoding EGFP protein alone (Panel **A** (a–c)) and EGFP-emerin fusion protein (Panel **A** (d–f) and Panel **B**). After 48 h, cells were stained for emerin, tubulin (Panel A) and also lamin A (Ac, B). Fluorescence of the EGFP alone and fusion protein was used to detect the location of a transfected protein (green). Panel **A** (a–c) demonstrates a typical, dispersed location of GFP protein in transfected cells in interphase and mitotis. Arrows indicate the location of centrosomes and mitotic spindle. Note (b,c) the location of membranes (visualized by emerin staining), emerin and tubulin in the center (b) indicated by an arrow with nice colocalization between emerin and tubulin while EGFP fills the entire space. See also Figure S2d for the similar location of emerin (and LAP2β) compared to membranes. Panel **A** (d–f) demonstrates typical distribution and level of the emerin-GFP fusion protein in transfected cells. In cells transfected with plasmid coding for the fusion protein EGFP-emerin (E1–254) staining for emerin and fusion protein signal (EGFP fluorescence) colocalizes nicely. In cells with a higher level of expression of a fusion protein, deposits of overexpressed emerin protein appear which recruit tubulin to these deposits if present at a high level (see arrowheads in Panel A, d). Note also frequent deformations of nuclear shape in cells overexpressing emerin fusion protein. At prophase (e,f) emerin is still located at the NE but a fraction of emerin associates with microtubules and centrosomes entering the nuclear space (arrows). Arrowhead points to one of the mitotic spindle microtubules associated with emerin (see also Panel **B**, d,e and Figure 3A, c for comparison). Panel B (a–e) demonstrates typical EGFP-emerin (E1–254) distribution together with lamin A and tubulin staining in mitotic cells. Arrows point to the location of centrosomes and spindle poles. Arrowheads point to the NE invaginations with emerin (d,e), which gradually lost its association with polymerized nuclear lamina as judged by weakening lamin A staining (see also Panel A, e,f and Figure 3A, c for comparison). Note the emerin fraction at the center of the nuclear space in Panel Bc and the disappearing fraction of internal lamin A in this space (see also Figure S2, d for comparison). Note the higher level of transfected emerin associated with mitotic spindle microtubules, which may result from the saturation of “normal” binding sites for emerin and competing for other binding sites. Single Z-section (1.0 μm) centered at centrosomes (mitotic spindle) is shown. Scale bar, 10 μm.

Studies of transfected fusion protein location are widely accepted as an efficient method of detection of the subcellular location of a particular protein, not burdened with potential problems associated with the specificity of antibodies. Therefore, we used this method to confirm our data obtained using the antibody approach. We used two plasmids for transient transfection encoding GFP alone and GFP-emerin fusion protein. Experimental conditions were set in such a way as to obtain a sufficient efficiency of transfection, in order to obtain sufficient amount of transfected mitotic cells, but without obtaining a too high level of expression (see Materials and Method section for details). Figure 4A, a demonstrates a typical view of transfected cells expressing GFP protein while Figure 4A, d illustrates a typical view of transfected cells expressing GFP-emerin protein in interphase. GFP-emerin fusion protein fluorescence fully colocalizes with staining for emerin (Figure 4B). Figure 4A, b,c demonstrates typical localization of GFP protein in metaphase cells. GFP protein fills in all possible free space in cells while staining for emerin and tubulin colocalizes and reflects the microtubule network associated with the mitotic spindle (Figure 4A, b, arrows). Figure 4A, c demonstrates a top view of the typical metaphase spindle and the lack of colocalization between GFP and tubulin staining. Based on the antibody staining, the level of exogenously delivered emerin does not exceed more than twice the level of endogenous protein (compare emerin staining in Figure 4A, a with Figure 4A, d). Interestingly, aberrant distribution of the tubulin network and the nuclear shape observed in transfected cells are not only associated with a high level of EGFP-emerin protein but are observed in cells with a comparable level of exogenous emerin. Note the tubulin aggregates associated with emerin deposits (Figure 4A, d arrowheads) in cells with different levels of emerin. Figure 4A, e,f demonstrates the typical early prophase of transfected cells in order to visualize centrosomes and microtubules entering the nuclear space and NE invaginations with emerin colocalizing with entering centrosomes and spindle tubulin (arrows, arrowheads). Note that Figure 4A, f demonstrates exactly the same phase of mitosis (prophase) as Figure 2A, a and demonstrates exactly the same location of endogenous emerin as in transfected cells and exogenous plus endogenous emerin. Note the microtubules connecting two centrosomes, visible in both figures (Figure 2 and Figure 4), which localize in the NE furrow nicely, seen also at early prometaphase (Figure 3A, c). Figure 4B demonstrates a different view of mitotic cells in order to demonstrate decoration of centrosomes and mitotic spindle microtubules with GFP-emerin protein. Note the perfect colocalization of GFP-emerin protein with centrosomes and microtubules of the mitotic spindle (Figure 4B, b, arrows) as well as with astral microtubules and colocalization of the lamin A fraction in the spindle area with emerin (Figure 4B, c, arrows).

### 3.5. Transient Transfection of Emerin Deletion Mutants Results in a Large Variety of Cellular Phenotypes

**Figure 5:**
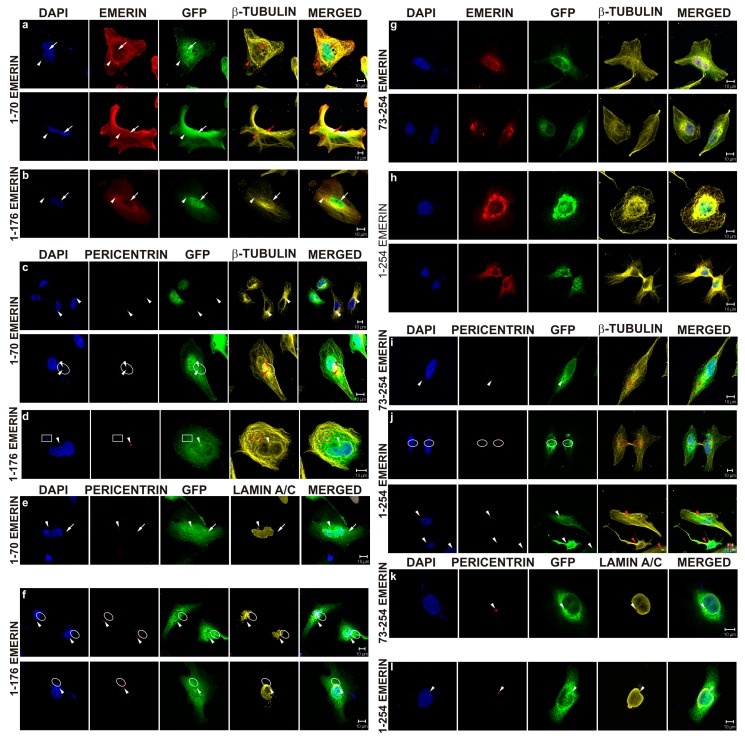
Overexpression of entire emerin and emerin fragments containing different domains in HeLa cells. Depending on the protein fragment introduced into the cell, we observed different effects manifested by disorder in the process of nuclear reassembly, maintenance of nuclear shape, and the microtubule network. For details, see main text. HeLa cells were transfected with plasmids coding for GFP fusion proteins (E70, E176, E73-254, or E254), placed on coverslips, fixed with 4% PFA, and stained with antibodies against emerin (red, **a**,**g**,**h**), pericentrin (red, **c**–**f**,**i**–**l**), β-tubulin (yellow, **a**–**d**, **g**–**j**), and lamin A/C (yellow, **e**,**f**,**k**,**l**). **a**,**b**: -arrows in a,b point the aberrant nuclei lacking endogenous emerin; arrowheads point the endogenous cytoplasmic emerin fraction. **c**–**l**: -arrowheads indicate the location of centrosomes; circles define the regions with multiple staining for pericentrin; boxes define the regions with extra nuclear DNA/chromatin. Single Z-stacks 1.5 μm. Scale bar, 5 μm.

Because our previous collaborative studies demonstrated a dominant-negative effect of emerin deletion mutants on in vitro nuclear assembly and NPC formation [10,52], we decided to use emerin deletion mutant constructs prepared in our laboratory (Figure 1) for transient transfection experiments to analyze the effect of a particular domain on cellular phenotype. Cells were transfected with plasmids under the same conditions for all constructs, in order to achieve the same plasmid copy number in transfected cells and the resulting protein level. All transfection reactions had similar transfection efficiency, and comparable levels of exogenous emerin protein were achieved, as determined using IF staining for emerin (Figure 5) and flow cytometry analyses (data not shown). Transfected cells with significantly lower or higher emerin protein expression, as judged by EGFP fluorescence and flow cytometry, were excluded from the analyses. Transfected cells were analyzed between 48 and 72 h after transfection to allow for exogenous protein expression but to prevent positive or negative selection of a particular phenotype among growing cells. Figure 5 shows the typical results of such experiments (see also Figure S3 for details of centrosome locations). Transient expression in HeLa cells of the E70 construct (GFP fused with the N-terminal 1–70 aa fragment of emerin) containing an intact LEM domain resulted in a variety of phenotypes of which the most frequently observed were irregularly shaped nuclei and, relocation of endogenous emerin to the cytoplasm while exogenous fusion protein located inside the nucleus and in the cytoplasm (Figure 5a,c,e). In some cells transfected by E70 and E176 mutants, we observed frequently fragmented nuclei and/or staining for chromatin/DNA outside cell nuclei (Figure 5a,c,d,f), which might suggest problems with chromosomes translocation by the mitotic spindle to daughter cells. We also observed the loss of a well-organized microtubule network and of pericentrin staining or dispersed staining for pericentrin, and atypical cell shape (Figure 5a,c,e). E70 mutant location was nucleoplasmic and cytoplasmic. E70 also affected the distribution of NL proteins (Figure 5e). The E176 construct (GFP fused with N-terminal 1–176 aa fragment of emerin) containing the LEM domain, most of the lamin A/C binding domain, and an intact tubulin binding domain also resulted in a cellular phenotype (Figure 5b,d,f). Irregularly shaped nuclei were less frequent than in the E70 mutant. E176 localized to the nucleus more distinctly than E70 and was present in the cytoplasm in diffused form and associated with microtubules. E176 also induced relocation of endogenous emerin into the cytoplasm, and its expression affected microtubule network organization and partially disconnected the centrosome from the network (Figure 5d) while inducing stronger staining for pericentrin in the centrosome or inducing dispersion of staining for pericentrin (Figure 5f). Relocation of E176 frequently affected the distribution of NL proteins, (Figure 5f).

Note that transfection of cells with E70 mutant (also frequently with E176 mutant) induced, besides irregular and fragmented cell nuclei, also the presence of chromatin loci in cytoplasm (Figure 5a,c,d).

The E73–254 mutant, lacking the entire LEM domain, localized predominantly to the cytoplasm and at the cytoplasmic side of the NE (Figure 5g,i,k) and relocated endogenous emerin to the cytoplasm and nuclear foci with a grain-like pattern or into large aggregates formed together with the E73–254 mutant. Endogenous emerin was also absent from the midbody, and very little E73–254 mutant protein localized there. The nuclear shape was only mildly deformed, but chromatin fragments occurred outside the nucleus (Figure 5k). The microtubule network was partly disorganized, with some tubulin staining outlining the nucleus. Centrosome location did not appear much affected in some cells (Figure 5k), but either a dispersed signal from pericentrin could be detected outside the centrosome or the centrosome was located much further apart from the cell nucleus within tubulin deposits (Figure 5i). The location of NL proteins was not visibly affected.

The E254 construct is a fusion protein of GFP and full-length emerin (Figure 5h,j,l). This fusion localized as endogenous emerin when expressed at low levels (see also Figure 4). Higher expression levels led to localization mostly outside the nucleus in big aggregates recruiting endogenous emerin. Endogenous and exogenous emerin colocalized perfectly. In some cells, the normal organization of the microtubule network was lost completely (Figure 5h), and emerin recruited microtubules to emerin deposits next to the NE. Emerin in the midbody was also lost in some cells. The localization of pericentrin appeared to be normal (Figure 5l), dispersed into a few or more spots or dispersed into many discrete spots and spanning the entire space with a high level of emerin in the cytoplasm (Figure 5j). NL proteins localized in a typical way (Figure 5l).

**Figure 6:**
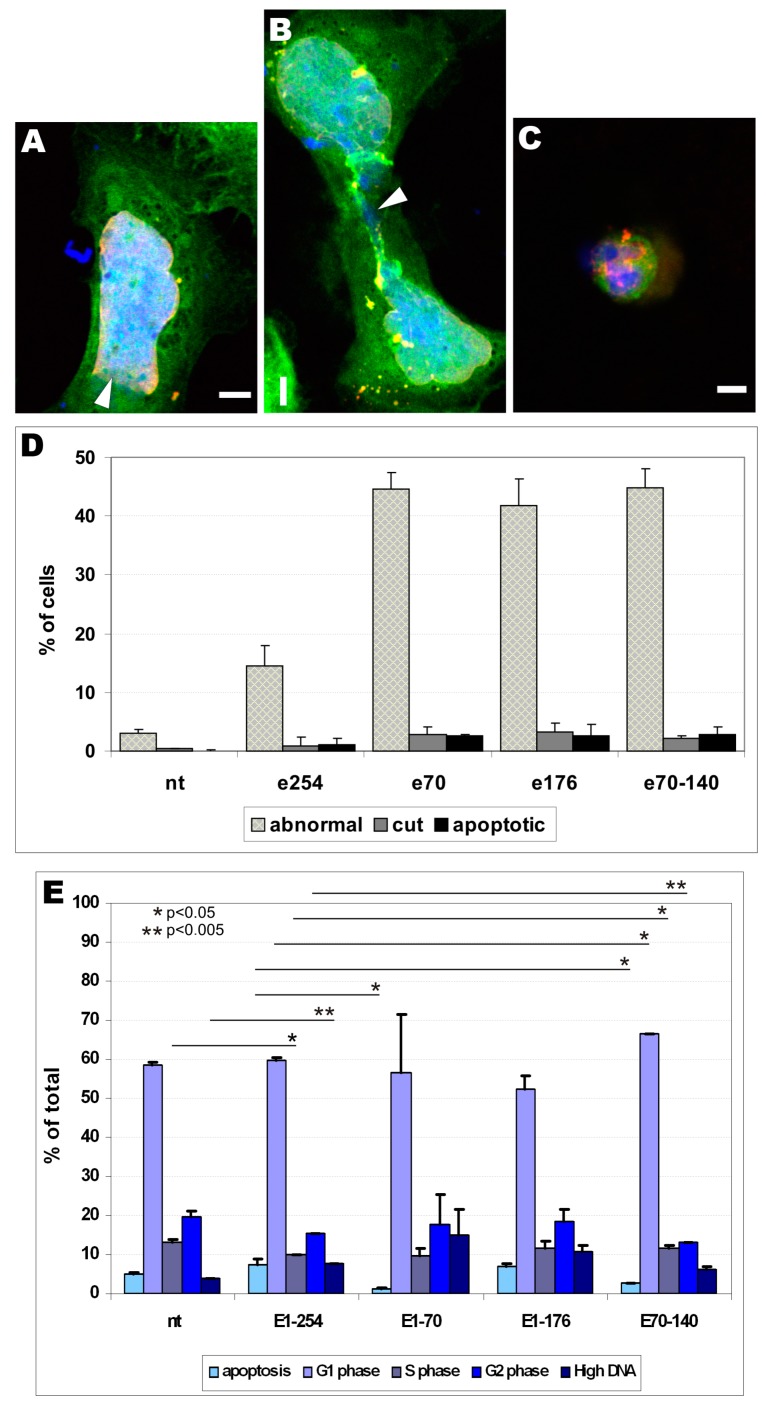
The effect of overexpression of emerin deletion mutants on the HeLa cell phenotype and cell cycle. Overexpression of emerin fragments induces the three most common atypical phenotypes, indicated as abnormal (**A**), “cut” (**B**), and apoptotic (**C**). Cells transfected with GFP-tagged emerin mutant (green) were stained for DNA (blue) and lamins A/C (red). Single confocal sections (1.5 μm) through the center of cells are shown. Bar, 5 μm. Only merged images are shown. (**D**) The results of manual counting and statistical analysis of phenotype frequency in untransfected cells (nt) and those transfected with the full-length emerin construct (E254) or emerin mutants (E70, E176, or E70–140). For the abnormal phenotypes, there were statistically significant differences (*t*-test < 0.05) between nt and all emerin constructs as well as for E254 and each emerin mutant. For “cut” and apoptotic phenotypes, only frequencies of nt versus all emerin constructs were significantly different. (**E**) Histogram showing cell cycle distribution for untransfected HeLa cells and those transfected with E254, E70, E176, or E70–140 constructs, based on flow cytometry data. Fixed cells were stained for propidium iodide and analyzed using flow cytometry. Apart from G1, S, and G2/M phases, low DNA (apoptotic) and high DNA (polyploidic) fractions also were distinguished. Overall, cells transfected with emerin mutants showed slightly decreased counts in G2/M phase, without increases in apoptotic cells.

To perform quantitative and statistical analyses of changes in cellular phenotype in transfected cells, we decided to define and count three easily detectable phenotypes: abnormal, cut, and apoptotic. Figure 6A–C and Figure S4 show representative phenotypes, along with typical staining for transfected fusion protein (EGFP fluorescence) and antibody staining for emerin (both endogenous and transfected protein) and lamins A/C and B1. An abnormal phenotype indicates an anomaly in nuclear shape and size. Often, it is also connected with the loss of NE/NL protein staining from one of the nuclear poles (Figure S4A). Two cells stacked at late telophase/cytokinesis with an abnormal midbody and atypical emerin/lamin network typify the cut phenotype, independently of their nuclear phenotype (Figure S4B, arrowheads). “Apoptotic phenotype” describes typical apoptotic nuclei with condensed cells with apoptotic bodies and an apoptotic nuclear lamina phenotype (Figure S4C).

Figure 6A–C shows the results of typical staining defining the abnormal (A), cut (B), and apoptotic (C) phenotypes, respectively. Cells transfected with EGFP-tagged emerin mutant E70 were stained for A/C type lamins and DNA. Figure 6D shows the frequency of a particular phenotype detected in transfected cells. Transient transfection of most of the constructs induced statistically significant changes in all three phenotypes compared to control cells. There was also a statistically significant difference between the E254 and all other tested emerin deletion mutants for abnormal phenotype. About 15% of cells showed an abnormal phenotype in E254 and more than 40% of cells in all other mutants, compared to about 3% in the control; however, none of these differences were statistically significant among the three phenotypes with E70, E176, or E70–140. No statistically significant changes were detected between control cells and cells transfected with E254 emerin with respect to the cut and apoptotic phenotypes (Figure 6D). No significant differences were detected for deletion mutants E70, E176, and E70–140 and E254 transfection regarding “cut” and “apoptotic” phenotype too. Thus, all of the emerin deletion mutants induced significant increases in frequencies of abnormal phenotypes while overexpression of E254 protein induced only an abnormal phenotype compared to the control.

Using flow cytometry, we performed cell cycle analyses together with counting apoptotic cells (low DNA content) and polyploidic (endoreplicating) cells (see methods for details). Figure 6E shows the results of the experiments. Transient expression of full-length emerin protein (E254) resulted in slightly increased apoptosis and cells with a high DNA content but a decreased number of cells in S and G2 phases, when comparing with untransfected cells.

All other analyzed mutants displayed variable levels of cells in a particular phase, and a few were statistically significant. In regards to E254 cell cycle distribution, the most significant changes were observed with the E70–140 deletion mutant, which showed a statistically significant increase in G0/G1 and S phases and decrease in G2 phase as well as for, high DNA and apoptotic fractions. Transfection of E70 resulted in a decreased number of apoptotic/sub-G0 cells but increased number of cells with high DNA content.

In general, transient expression of deletion mutants favored cells to choose quiescence, rather than apoptosis, along with minor cell cycle phases differences.

### 3.6. Full Nucleoplasmic Domain of Emerin can Bind Chromatin Condensed Chromatin Independently of BAF, Membranes, and Tubulin

The overall result of this part of the study is that the presence of an emerin deletion mutant or exogenous wild-type emerin protein resulted in statistically significant, readily detected phenotypes associated with the nuclear structure, centrosome location, and various microtubule phenotypes. Since some of the deletion mutants were also able to block in vitro nuclear assembly in the *Xenopus* oocyte extract system [10,52] we decided to analyze the chromatin binding ability of the particular deletion mutant protein and possibly the potential type of interaction that might be affected in a particular mutant. The tests of the functionality of the deletion mutants have already been described [52], using the *Xenopus* in vitro nuclear assembly reaction, and indicated that some of the deletion mutants were dysfunctional and acted as dominant-negative mutants, especially E70. In another study testing the effect of emerin on centrosome location and interaction with microtubules, the E176 fragment displayed the highest affinity [62]. This deletion mutant could bind tubulin, in contrast to E70, E70–140, and E70–176. Thus, the domain responsible for centrosome mislocation and tubulin network misorganization can be pinpointed as that associated with the E176 deletion mutant.

Based on this inference, we tested the chromatin binding properties of emerin deletion mutants expressed and purified from bacteria (Figure S5A,B). For this purpose, we used the simple chromatin binding assay from *Xenopus* [69]. Figure S5 illustrates the chromatin binding ability of bacterially expressed emerin deletion mutants. As a control we used already tested proteins from *Xenopus*: N-terminal LAP2 fragment (nucleoplasmic, common fragment) [54,55,57,58,69] and the N-terminal, nucleoplasmic LBR fragment [71,72]. *Xenopus* LAP2 protein represents chromatin binding through the LEM domain and BAF protein [73,74,75], while the N-terminal LBR fragment interacts with chromatin directly and through HP1 via the Tudor domain and chromodomain, respectively [72,76,77]. Figure S5C demonstrates that bacterially expressed BAF protein binds to condensed sperm chromatin and to decondensed chromatin in Pfaller buffer (with poly-glutamic acid). Moreover, cytosol-mediated decondensation (and possibly modification by cytosol) increased binding. E70 and E176 deletion mutants did not bind chromatin, while the E220 deletion mutant lacking only the transmembrane domain and the very C-terminal (luminal) portion of the protein bound condensed and de-condensed chromatin efficiently. XLAP2 protein, containing the so-called common domain of XLAP2 isoform (with a LEM domain and probably at least part of a lamin-binding domain) fragment, bound inefficiently to condensed chromatin, whereas decondensation by the cytosolic fraction increased binding. Similarly, the nucleoplasmic domain of XLBR protein, containing the HP1 binding site and Tudor domain for direct chromatin binding, bound inefficiently to condensed chromatin and very efficiently to decondensed chromatin. These data indicate that neither the LEM domain alone (E70) nor the LEM domain with lamin binding/tubulin binding domains (E176) can bind to chromatin but that the entire nucleoplasmic domain (E1-220) can bind efficiently to all chromatin forms in the absence of the membrane fraction. Our data correlate with already mapped regions of interactions of emerin, XLAP2, and LBR proteins [11,52,57,58,60,71,72,73,76,77,78,79]. Our data suggest that for bacterially expressed emerin to assemble at chromatin simple decondensation of chromatin is sufficient and no prior modification of chromatin or the membrane fraction is required as in the case of the bacterially expressed XLBR nucleoplasmic fragment.

### 3.7. Detailed Analyses of Emerin Protein Expression in Patient Cells Reveal Truncated Emerin Protein Presence and an Epitope Masking Effect in Immunofluorescent Analyses

**Figure 7:**
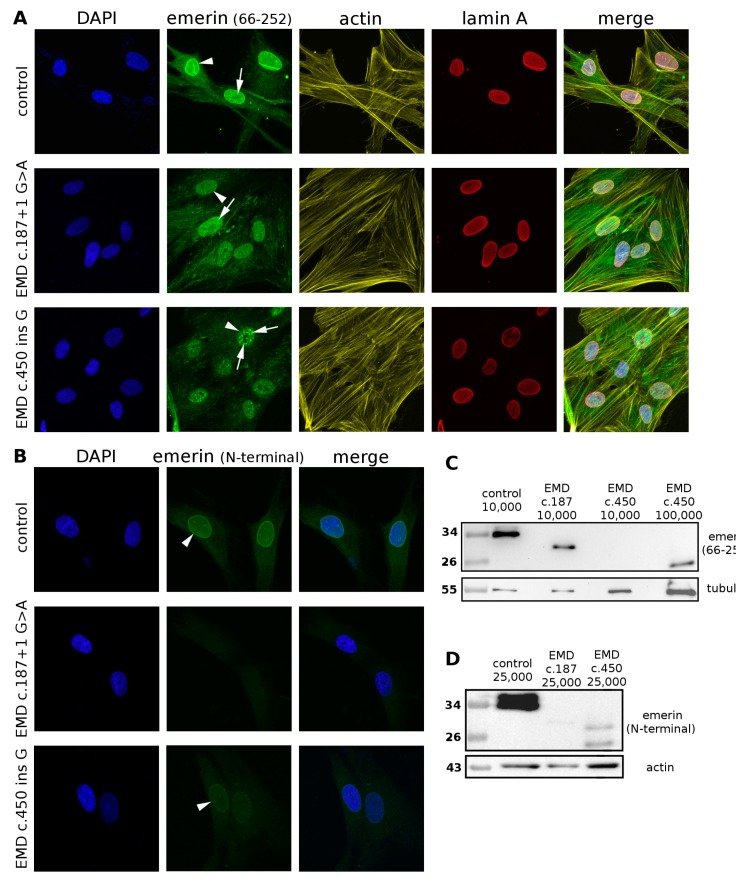
Selected different “emerin-null” patient cells express emerin mutant protein and show abnormal emerin location. Emerin distribution and presence in NHDF and fibroblast obtained from patients with EDMD1. EDMD1 patient cells have shortened emerin with abnormal localization or lower levels. (**A**) Immunofluorescence staining with antibodies recognizing 66–254 aa of emerin indicates changes in the distribution of this protein (emerin diminishing in nuclear rim) in patients bearing mutations c.187 + 1G > A (P2) and c.450insG (P6) in comparison to normal cells. Mutants of emerin do not affect the shape of nuclei (staining for lamin A) or actin filament structure. (**B**) Staining with antibodies recognizing the N-terminus end of emerin indicates lack of epitope in the N-terminal fragment in a patient sample with the mutation c.187 + 1G > A (P2) and shows proper but weaker staining of emerin in the nuclear envelope in a patient sample with the mutation c.450insG (P6), as in the control; the difference in staining pattern between these two antibodies may be because of epitope availability [80] (**C**) Western blot analysis using antibodies recognizing 66–254 aa of emerin shows the presence of truncated proteins in both patients; the level of emerin is lower than in the control. (**D**) Western blot analysis using antibodies recognizing the N-terminal end of emerin shows the low level of truncated proteins in a c.450insG (P6) patient sample; emerin is not detected in a c.187 + 1G > A (P2) patient sample.

In fibroblasts with silenced emerin expression as well as emerin-null fibroblasts from patients with EDMD1 (*EMD* del153C), centrosomes are relocated from the nuclear envelope [62]. Therefore, we tested several fibroblast cell lines established from Polish patients with EDMD1, all cases believed to be emerin-null based on immunofluorescence analyses. We selected several cell lines from patients with the following mutations: P2: c.187 + 1 G > A; P3 and P4: c.del153C; P5: c.399 + 1G > C; and P6: c.450insG. Thorough but preliminary IF and Western blot analyses using a variety of our self-made antibodies and commercial antibodies allowed us to detect a truncated emerin protein in the P2 and P6 mutant lines (Figure 7). IF analyses of control and patient cells using one of the available commercial anti-emerin antibodies specific for almost the entire emerin sequence (66–254 aa, without the N-terminal LEM domain) revealed emerin in nuclei (Figure 7A).

Staining of the same cells with N-terminal-specific antibodies from a different manufacturer (see methods) identified emerin in control and P6 fibroblasts, but not in P2 samples (Figure 7B). Staining for emerin (Figure 7A) shows a different pattern in control and patient cells. In controls emerin is detected in the NE (arrowhead) and a few foci inside the nucleoplasm (arrows). The nucleoplasmic and cytoplasmic fraction is very small. In P2 fibroblasts, staining of nucleoplasm is dominant, with many intranucleoplasmic foci (arrow), no staining for NE is observed and increased staining in the cytoplasm is detected (Figure 7B). In patient P6’s fibroblasts emerin is detected in the nucleoplasm with stronger but fewer foci (arrow) and no labeling of NE while the cytoplasm was stained at a similar level. Western blot analyses performed with the same antibodies as described in Figure 7A showed the presence of truncated emerin protein in patient cells carrying the P2 or P6 mutation (Figure 7C). The level of truncated emerin was lower than that of wild-type emerin in control and P6 mutant cells but at the level detected by antibody. Protein was visualized only when higher amounts of cell extracts were loaded per lane (Figure 7C,D). Western blot analyses of cells using N-terminal-specific antibody detected nothing in the P2 mutant cells (Figure 7D), similar to the results of the IF analyses (Figure 7B). The P6 emerin mutant protein was present at very low levels compared to the control (Figure 7C,D), so very little was detected with the N-terminal-specific antibody in IF (Figure 7B).

Our findings suggest that the presence of truncated emerin protein, although at a fraction of the original level, containing only the N-terminal fragment with the LEM domain, may contribute to the severity of disease phenotype by a toxicity effect similarly to the E70 deletion mutant in our transfection studies.

## 4. Discussion

### 4.1. Subcellular Location of Emerin during Mitosis: Disassembly and Reassembly

The initial studies of emerin location in cells were performed mainly to demonstrate the presence of the protein and the location of the protein inside the cell nucleus. Therefore, only a few data were available demonstrating emerin location during the cell cycle. Dabauvalle and coworkers provided fluorescent microscopy image of emerin and tubulin which might suggest the possible colocalization of the emerin fraction with microtubules during mitosis (Figure 4 in original paper [59]). Live fluorescent microscopy studies performed by Haraguchi and coworkers provided the first GFP-emerin studies during anaphase and telophase and demonstrated emerin fusion protein associated at decondensing chromatin in live cells [73]. The same team using live study demonstrated GFP-emerin association with “core regions” of decondensing chromatin during anaphase and showed that GFP-emerin lacking the LEM domain associates with the entire decondensing chromatin, not only with “core regions” [11]. Another study by the same team, combining time-lapse fluorescent microscopy with TEM, using GFP-fusion proteins, demonstrated colocalization of all major nuclear karyoskeletal proteins during anaphase and telophase with BAF, microtubules and membranes at the decondensing chromatin surface [61]. They also demonstrated, in a separate experiment, the localization of GFP-BAF protein to the metaphase and telophase mitotic spindle in fixed cells (see Figure 10 in original paper [61]), but this location of BAF was not detected in live fluorescent microscopy. This might suggest that time-lapse microscopy studies not focused on a particular cell feature or process may not provide enough data to discover less obvious phenotypes or when the proper question is not asked. For example, the study demonstrating that emerin localizes in the INM and ONM [62] was published relatively late simply because no one asked the question about emerin location on the cytoplasmic face of NE before. There was a similar situation with the intranuclear lamin A/C fraction [81]—no one reported intranuclear location of lamin A/C although hundreds of studies had been performed and published on lamin A/C by then. In this study, focused solely on mitotic distribution of emerin using laser confocal microscopy, we demonstrated the fraction of emerin associating with tubulin of the mitotic spindle and centrosomes. Our data demonstrated colocalization of the emerin fraction with polymerized tubulin during mitosis (Figure 2A), and it resembles the emerin and tubulin distribution from the study by Dabauvalle et al. [59] except that we obtained better quality and resolution in our multichannel confocal study. Interestingly, we also found colocalization of the endogenous BAF fraction with the mitotic spindle (Figure 2B) through the entire mitosis, which was suggested by GFP-BAF localization to the spindle during metaphase and telophase [61]. We also demonstrated that during early mitosis (prophase, prometaphase), microtubules and centrosomes entering the nuclear space are accompanied by NE membranes with integral proteins (e.g., emerin) and nuclear lamina fragments not entirely depolymerized at this time (Figure 3). By staining the membranes with DHCC dye, together with immunostaining for emerin and the mitotic spindle (Figure S1), we demonstrated that emerin associated with the mitotic spindle and centrosomal tubulin is still associated with membranes. We also demonstrated the colocalization of a fraction of another integral membrane protein of INM-LAP2β with membranes and the mitotic spindle (Figure S2). These results suggest that during prophase and prometaphase, the forming mitotic spindle and centrosomes with microtubules entering the nuclear space associate with the fraction of integral membrane proteins such as emerin and LAP2β, BAF protein, membranes and not fully depolymerized nuclear lamina. The existence of such a fraction and such a mechanism of retaining nuclear lamina and membrane proteins at the mitotic spindle might facilitate the nucleus reassembly mechanism.

This phenomenon might represent exactly the same but inverted mechanism governing the nuclear reassembly when karyoskeletal proteins, integral membrane proteins, such as emerin and LAP2β, and membranes are deposited via microtubules through BAF interaction with chromatin at core regions during anaphase [61,67,70,74,82] One part of the generally accepted mechanisms regulating nucleus and chromatin reassembly depends on BAF chromatin interactions [11,36,61,67,74,83] and the BAF reactivity depends on phosphorylation status since phosphorylation by VRK1 kinase disrupts its interaction with chromatin while knockout of VRK1 kinase leads to BAF retention on mitotic chromosomes [84]. Dephosphorylation of BAF at the end of mitosis depends on the interplay between LEM4 protein (ANKLE2), which is a cytoplasmic, ER-located integral membrane LEM-domain protein, PP2A phosphatase and VRK1 kinase [85,86]. PP2A phosphatase, associated with LEM4 at the ER membrane during mitosis, dephosphorylates BAF, which is then able to bind to chromatin and other LEM domain proteins from the INM. LEM domain proteins (with BAF protein), anchored in membranes, are delivered on microtubules to core regions of decondensing chromatin and deposited there. Lack of BAF dephosphorylation should also lead to aberrant location of other LEM domain proteins, such as the LAP2 family of proteins including MAN1, and indeed LEM4 depleted cells demonstrate aberrant reassembly of LAP2β protein [86]. Prolonged mitosis was observed and LAP2β protein associated outside core regions. Note that in live analyses, emerin lacking the LEM domain or a LEM domain mutant (not interacting through BAF) localizes to the entire surface of decondensing chromosomes, not to core regions [11]. In LEM4 depleted cells, emerin reassembled at the mitotic chromatin also much later and assembled with the same efficiency with the entire chromatin—not only at core regions. This suggests that this might be an alternative assembly mechanism not dependent on BAF/LEMD4/microtubule/membranes which deposits emerin and LAP2β proteins at core regions [86]. Since BAF not phosphorylated by VRK1 kinase still associates at mitotic chromosomes [85], we may assume that binding sites for BAF on mitotic chromosomes are preserved. This perfectly agrees also with uniform binding on decondensing chromatin of emerin protein without the LEM domain [11]. Interestingly, LEM4 depletion leads to prolonged mitosis and aberrations in phenotype after mitosis, similarly to previous live studies on emerin [11,61], but cells are able to reassemble the cell nucleus [86] together with BAF, emerin and LAP2β, which suggests that there must be an alternative pathway or rescue mechanism allowing for removal of the mitotic pattern of phosphorylation on BAF and LEM-domain proteins of the INM (such as emerin and LAP2β). Additionally, there are several, independent of BAF, mechanisms of nucleus reassembly after mitosis which depends on other proteins and interactions. The obvious ones are LBR protein–chromatin and LBR protein–HP1 protein interactions, interaction with chromatin through NETs, LAP1, all interactions through other nuclear lamina proteins, etc.

In order to confirm our studies and to provide comparable data to previous studies on emerin we decided to analyze fixed cells as allowing for more precise and detailed study of discrete protein location compared to live fluorescent imaging [61] and providing an extra amount of protein which may interact with increased efficiency with binding partners and typically slowing the cell cycle [87]. As a control for the unspecific location of neutral protein we used GFP protein. As expected, GFP filled all available space in cells while GFP-emerin fusion protein location reflected the distribution of endogenous protein (Figure 4), although the fraction of GFP-emerin associated with mitotic spindle microtubules was proportionally higher. A similar effect was observed earlier for GFP-BAF visualization in HeLa mitotic cells at metaphase and telophase in fixed cells [61] and no staining was detected during live imaging. Interestingly, using GFP-emerin fusion protein and immunostaining it was possible to demonstrate the perfect colocalization of endogenous with exogenous fusion emerin protein (Figure 4). It was also possible to repeat exactly the same phenotypes of emerin and lamin A/C location during mitosis as obtained on untransfected cells. The only difference between immunofluorescence studies and transfection studies was the increased fraction of emerin associating with tubulin during mitosis, which might be explained by the presence of an extra amount of protein available for interactions. As mentioned above, a similar effect of transfected protein was previously observed for other proteins compared to the endogenous one [61,88,89].

Currently presented data on in vitro chromatin binding by the emerin deletion mutant (Figure S5) nicely correlate with phenotype development in transfected cells and with previous data on in vitro assembly. The strongest nuclear phenotype was detected with the E70 mutant, which is unable to bind to chromatin in absence of BAF and blocks the reassembly pathway through BAF docking sites at chromatin and relocating endogenous emerin. The E176 mutant also cannot bind to chromatin in vitro, inhibits reassembly mechanisms mediated by BAF protein, and also relocalizes endogenous emerin and affects the tubulin network since it contains a tubulin-binding domain (see Figure 1). Interestingly, the E220 mutant (full nucleoplasmic domain) can bind to chromatin independently of whether it is or is not decondensed, similarly to the nucleoplasmic domain of XLAP2 protein, which suggests a different assembly mechanism of chromatin binding than through BAF. Moreover, BAF protein binds to any form of chromatin. Note that the XLBR fragment (full, N-terminal nucleoplasmic domain) also binds to any form of chromatin as expected, since LBR does not interact with BAF protein during reassembly. These chromatin binding data provide information that bacterially expressed BAF binds any chromatin and since BAF is not phosphorylated, confirms its binding ability to chromatin of any form. This experiment also supports our suggestion regarding alternative mechanisms of emerin and LAP2 independently of interaction with BAF, since we demonstrated that full nucleoplasmic domains can associate with chromatin and decondensation only increased binding, so in vivo emerin and LAP2 can associate with decondensing chromatin independently of BAF. Therefore, in LEM4-depleted cells, emerin and LAP2 can assemble to chromatin but by mechanisms independent of dephosphorylated BAF protein. Alternatively, dephosphorylation of BAF can take place via a mechanism alternative to LEM4/PP2A at late telophase/early G0 stage or both mechanisms may be active.

Analyses of distribution of membranes, emerin, and LAP2β during mitosis indicated that a fraction of membranes, LAP2β, and emerin associates with spindle microtubules (Figure S2). Interestingly, we observed discrete differences in location of emerin and LAP2β with respect to the membrane fraction and tubulin. LAP2β distribution inside the nuclear compartment during prophase strictly followed the membrane distribution (Figure S2, arrowheads), while emerin distribution was more reflective of centrosome and microtubule location (arrows) and the association with membranes was barely visible. Note that intensity of staining for both portions was comparable at the nuclear envelope at interphase. These data might suggest the presence of a discrete fraction of membranes or domains in membranes, which has been implied earlier in the *Xenopus* in vitro nuclear assembly system [52] and a tissue culture model [90] but has also been refuted [91].

### 4.2. Emerin, LAP2β, Lamin A/C, and Membrane Location of Mitotic Spindle Microtubules

Our previously published collaborative data indicated that emerin deletion mutants inhibit in vitro nucleus assembly, chromatin decondensation, and nuclear pore complex assembly in a *Xenopus* system [52], similar to the XLAP2 protein in comparable studies [11,38,54]. One study reported that emerin fragments 73–180 and 1–176 bind to polymerized tubulin/microtubules [62]. Knockout of emerin in fibroblasts or fibroblasts from emerin-null patients with EDMD1 results in mislocation of centrosomes [62]. Emerin interacts with many proteins during interphase [34,35], among which LUMA [41], HDAC3 [42], Btf [43], GCL [44], actin [45], Lmo7 [46], NET25 [47], and β-catenin [48] appear most crucial in the context of the structure of the cell nucleus, nuclear lamina, and chromatin. Emerin interacts with Samp1 (NET5) [92] and is indirectly involved in positioning of two chromosomes in the cell nucleus [93], but previous studies have also found no changes in location of chromosome territories in emerin-null patients [94].

These data and interactions taken together suggest the direct involvement of emerin in nuclear reassembly mechanisms mediated through the LEM domain and a BAF-dependent pathway [38,60,78,95]. Therefore, identification in a tissue culture model of the fraction of emerin associated with mitotic spindle microtubules and centrosomes confirms the earlier reported interaction with tubulin. It also suggests an additional role for emerin during mitosis, which might be tethering a fraction (subpopulation/subdomains) of nuclear membranes to spindle microtubules. Of interest, a similar fraction of another LEM domain, lamin A/C-interacting, integral membrane protein-LAP2β, also associates with the mitotic spindle. Based on our analysis of the discrete pattern of the mitotic fraction of emerin and LAP2β, we can say that although both proteins associate with membranes, their locations differ slightly: emerin (plus associated membranes) follows microtubules and centrosomes at prophase and prometaphase while LAP2β associates perfectly with (bulk/total) membranes. Because emerin and LAP2β show discrete locations at the mitotic spindle, this pattern suggests various mechanisms for retaining a fraction of integral membrane proteins, with membranes, to the mitotic spindle elements based on their specific interaction. The existence of at least two populations of membranes with different composition and function was demonstrated in *Xenopus* in vitro assembly, where an exogenous emerin deletion mutant inhibited binding only of one membrane fraction [52]. Inhibition of binding inhibited assembly of Nup153 and other internal, nuclear nucleoporins, such as p62, but not of Nup358 and Nup214.

This discovery of membrane populations associated with a specific integral membrane protein (emerin versus LAP2β membranes) also revives the old discussion about the fate of NE membranes and INM integral proteins during mitosis in a tissue culture model [90,91,96]. Discrete and separate locations of emerin and LAP2β seem to favor the specific-membrane domains model.

Emerin and LAP2β are not unique nuclear proteins associated with the mitotic spindle. Despite Samp1 (NET5), some reports also suggest the existence of a spindle envelope and spindle matrix structure composed of B-type lamins [13,97,98,99]. These reports are in agreement with our data showing that lamins A/C and membranes also colocalize and associate with the mitotic spindle. Association of essential structural proteins with the mitotic spindle might be a “clever” evolved mechanism, similar to chromosomal passenger protein transfer, ensuring the availability of proteins necessary to decondense and reassemble chromatin after mitosis. Microtubule movement or microtubule motor movement might be among the mechanisms delivering proteins and membranes to their destination at chromatin/chromosomes. Preferential location of emerin (and LAP2β) at core regions of decondensing chromosomes and colocalization of emerin and tubulin at microtubule–chromatin contact sites favor such a mechanism (see section above). Similar dense regions can be seen for LAP2β protein at core regions while BAF and lamin A/C, although also associated with the mitotic spindle, showed smooth and uniform staining at anaphase.

### 4.3. Role of Emerin Domains

As we expected, overexpression of emerin deletion mutants in HeLa cells frequently induced abnormal phenotypes, including changes in nucleus shape and NE/NL structure, lamin localization, microtubule and centrosome distribution, and sometimes redistribution of ER membranes. These changes appeared in variable magnitudes, frequencies, and combinations, so we selected only three easily detectable and countable phenotypes for analyses: abnormal, cut, and apoptotic. Of interest, simple transient expression of full-length emerin protein increased the frequency of the abnormal phenotype, indicating that not only the dysfunctional protein but also the ratio of the protein to interacting partners may be critical. Expression of the LEM domain alone (E70), the LEM domain with lamin A/C binding domain (which perfectly correlates with tubulin binding domain E176) [62], or part of the lamin-binding domain alone (E73–140) resulted in the highest frequency of phenotype appearance. The implication is that all of the expressed mutants are dominant negative and none of them support the full interactions necessary for proper emerin function.

This result correlates with the in vitro nuclear assembly data and chromatin binding assay in which only the full nucleoplasmic domain of emerin could bind to any form of chromatin, while E70 and E176 mutants could not bind to chromatin at all. The control proteins BAF, XLAP2 fragment, and XLBR fragment do bind to decondensed and condensed chromatin, and E70 blocks in vitro nuclear reassembly [62] while E73–180 does not. Because in the in vitro system, E70 blocks both decondensation and assembly of the intranuclear nucleoporins Nup153 and p62 but not Nup214 and Nup358 [52], it is plausible to conclude that emerin may affect basket assembly and linkage between NPCs and nuclear lamina, as Nup153 supports interaction with the nuclear lamina via B-type lamins [100]. At the same time, E70 blocks decondensation by blocking NEP-A and NEP-B vesicles.

In a tissue culture model, the mechanism of phenotype development might be much more complex. E70 action results in saturation of free docking sites on BAF/chromatin, possibly blocking all LEM-domain protein–BAF–chromatin interactions [37,38,40,53,57,58,74,75,101,102] but not affecting other pathways (e.g., LBR-histones/DNA or LBR-HP1, or NETs-dependent). The E176 mutant acts in a similar way and additionally, through lamin-binding domain and tubulin-binding domain may saturate some of the lamin A/C binding and tubulin binding. Although recent reports cite the existence of trimeric complexes among BAF, emerin, and lamin A and the need for emerin dimerization [36,40], these findings do not preclude direct BAF–emerin LEM domain interactions [11,103]. The dominant-negative activity of the E70–140 emerin mutant seems to be a mystery. According to published data and our knowledge, this fragment should not possess any functional activity on its own; therefore, we might speculate that it may inhibit wild-type emerin interactions, block the dimerization of wt emerin, block the biological functions of emerin binding protein(s), or a combination of these effects.

Thus, in our tissue culture model, E70 and E176 induced a nuclear phenotype and frequent mislocation of centrosomes and the tubulin network while LEM domain deletion mutants affected mostly the cytoplasmic phenotype. These findings imply that in a tissue culture model, for proper nucleus reassembly, a functional LEM domain, together with a lamin A/C binding domain and dimerization domain (E220), is necessary, but for the wild-type cytoplasmic phenotype, the full-length protein is necessary.

### 4.4. Pathogenetic Mechanisms in EDMD1 Patients

Although mutations in the human *EMD* gene are most closely tied to the genetic disorder EDMD1, similar phenotypes arise from mutations in genes coding for interacting proteins, such as *LMNA* [104], *SYNE1*, *SYNE2* [105], and *TMEM43* [106]. Thus, missense mutations in *EMD* are thought to inactivate particular domains and affect interactions with binding partners as the molecular background of the disease. The involvement of these proteins in EDMD1 suggests that the main cause of disease is disturbance of nuclear integrity, stability, and signal transduction through the nuclear envelope. The other possible mechanisms of EDMD1 may be connected with disturbance of E2F-dependent gene expression, Btf-dependent transcription repression, the Wnt/β-catenin pathway, or nuclear corepressor complex-dependent chromatin modification (reviewed by [2]).

A recent report showed that in laminopathies, especially in EDMD1, we might face some discrepancy in misidentification of so-called emerin-null patients, and some emerin-null phenotype mutations (e.g., c.450 − 2A > G) are misdiagnosed because of improper selection of emerin antibodies [107]. In addition, in emerin (and lamin B1 and B2) detection using IF, epitope masking might be a serious problem in EDMD1 diagnosis and detection of proteins in tissue sections [80].

With samples from a group of Polish patients, we confirmed in preliminary studies that some of the analyzed mutations result in expression of truncated emerin protein. Moreover, electrophoretic mobility of truncated emerin corresponded to the predicted open reading frame generated by the mutations. Such a truncated emerin protein could act as additional element of development of severity of phenotype in patient cells, as we demonstrate here.

## Supplementary Materials

The following are available online at https://www.mdpi.com/2073-4409/8/3/240/s1, Figure S1: Cell cycle–dependent distribution of membranes, TPX2 and emerin in HeLa cells, Figure S2: Cell cycle–dependent distribution of membranes, LAP2, and emerin in HeLa cells, Figure S3: Transient transfection of HeLa cells with emerin deletion mutant constructs affects the location of centrosomes and tubulin network, Figure S4: Demonstration of typical phenotypes classified as “abnormal”, “cut” and “apoptotic” induced by expression of emerin deletion mutants using E70 mutant as an example, Figure S5: Xenopus sperm chromatin in vitro binding assay of chromatin association with emerin deletion mutants and other well-known chromatin-binding nuclear lamina proteins.
