# Supplementary material for: Emerin Is Required for Proper Nucleus Reassembly after Mitosis: Implications for New Pathogenetic Mechanisms for Laminopathies Detected in EDMD1 Patients
Source: Cells. 2019 Mar 13;8(3):240. doi: 10.3390/cells8030240 (PMC6468536; doi:10.3390/cells8030240)
Supplement: Supplementary file 1 [file cells-08-00240-s001.pdf]

Supplementary Figures:

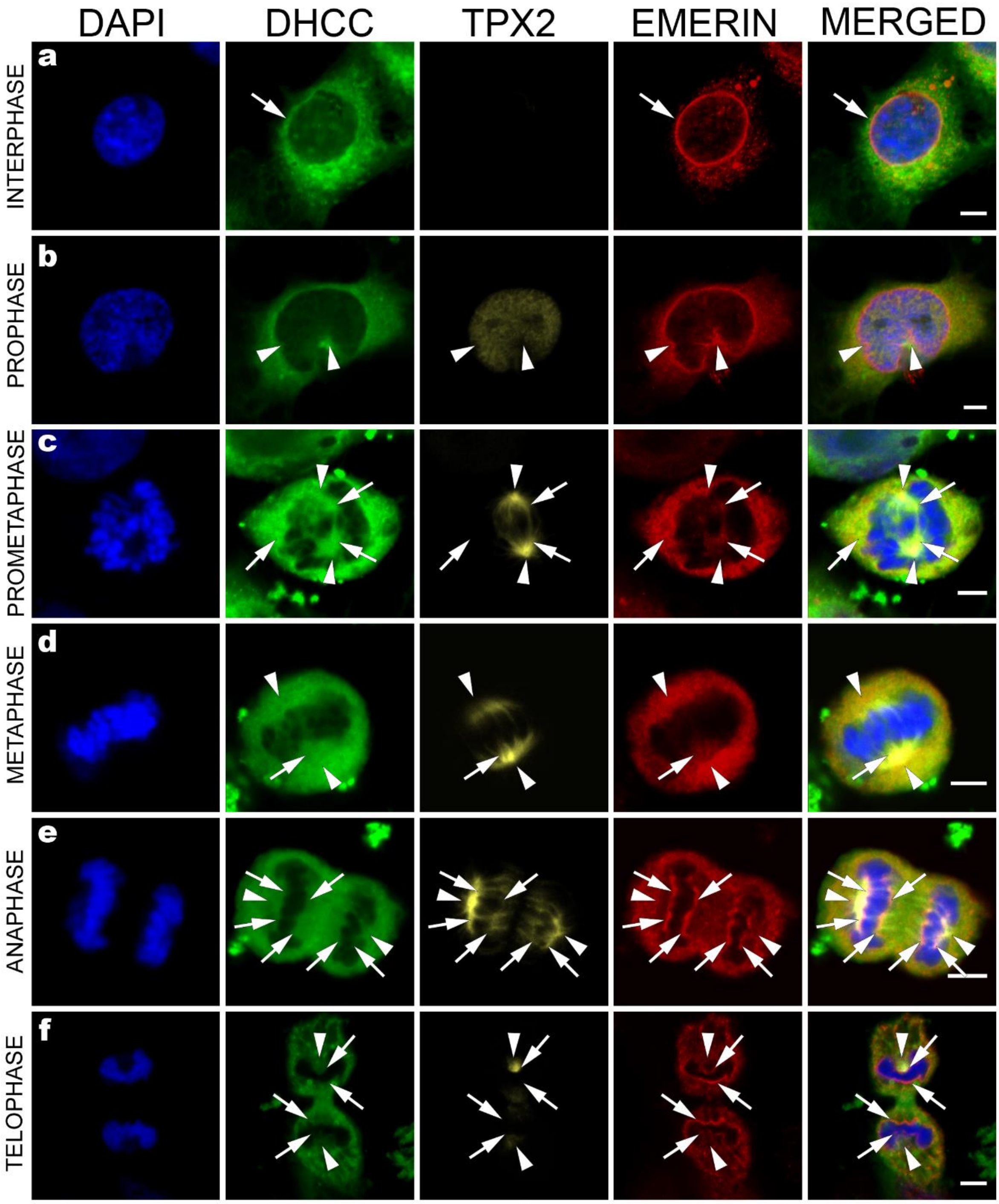

Figure S1. Cell cycle-–dependent distribution of membranes, TPX2 and emerin in HeLa cells. Cell cycle-–dependent distribution of emerin and membranes with respect to TPX2 (microtubule nucleation factor) in HeLa cells. Fractions of both membranes and emerin remain in the centrosome and mitotic spindle area during mitosis. a – in interphase emerin is concentrated in nuclear envelope (arrow); b – in prophase, the "centrosomal" fraction of emerin and membranes appears despite the early stage of mitotic microtubule formation (b). Note the two invaginations of the NE forming by centrosomes and microtubules entering the nuclear space (arrowheads) ; c, d – in prometaphase and metaphase the bulk fraction of membranes and emerin localizes in the cytoplasm, and only a fraction of membranes localizes at the mitotic spindle and centrosomal regions (c, d, arrows) (c, d arrows).

Although membranes are visualized at the mitotic spindle location from prophase to metaphase, emerin only partially colocalizes with membranes at the mitotic spindle, following mainly spindle microtubule location. At metaphase a fraction of membranes colocalizes with a fraction of emerin and mitotic spindle (arrow). e – in anaphase similar colocalization between fractions of membranes and emerin at the mitotic spindle is visible , also at both sides of decondensing chromosomes, but with higher density on the centrosome's side (arrowheads). Note also the higher density of emerin staining at chromatin loci colocalizing with microtubules (arrows). f – in telophase, membranes are almost equally distributed through the dividing cell, also retaining location of centrosomal regions and at the surface of decondensing chromatin. A similar distribution of emerin with higher density at the decondensing chromatin is observed (arrows). Note more diffuse staining for emerin on chromatin at the centrosomal face and emerin staining surrounding the centrosome in the upper nucleus (arrowheads). HeLa cells were grown on coverslips, fixed with 4% PFA, and stained for emerin, TPX2, and membranes (DHCC). DNA was visualized with DAPI. Single confocal sections (1.5 μm) through the nucleus but centered at centrosomes or mitotic spindle are shown. Arrowheads indicate the position of centrosomes visualized during later stages of mitosis (c-f) by TPX2 staining. Bar, 5 μm.

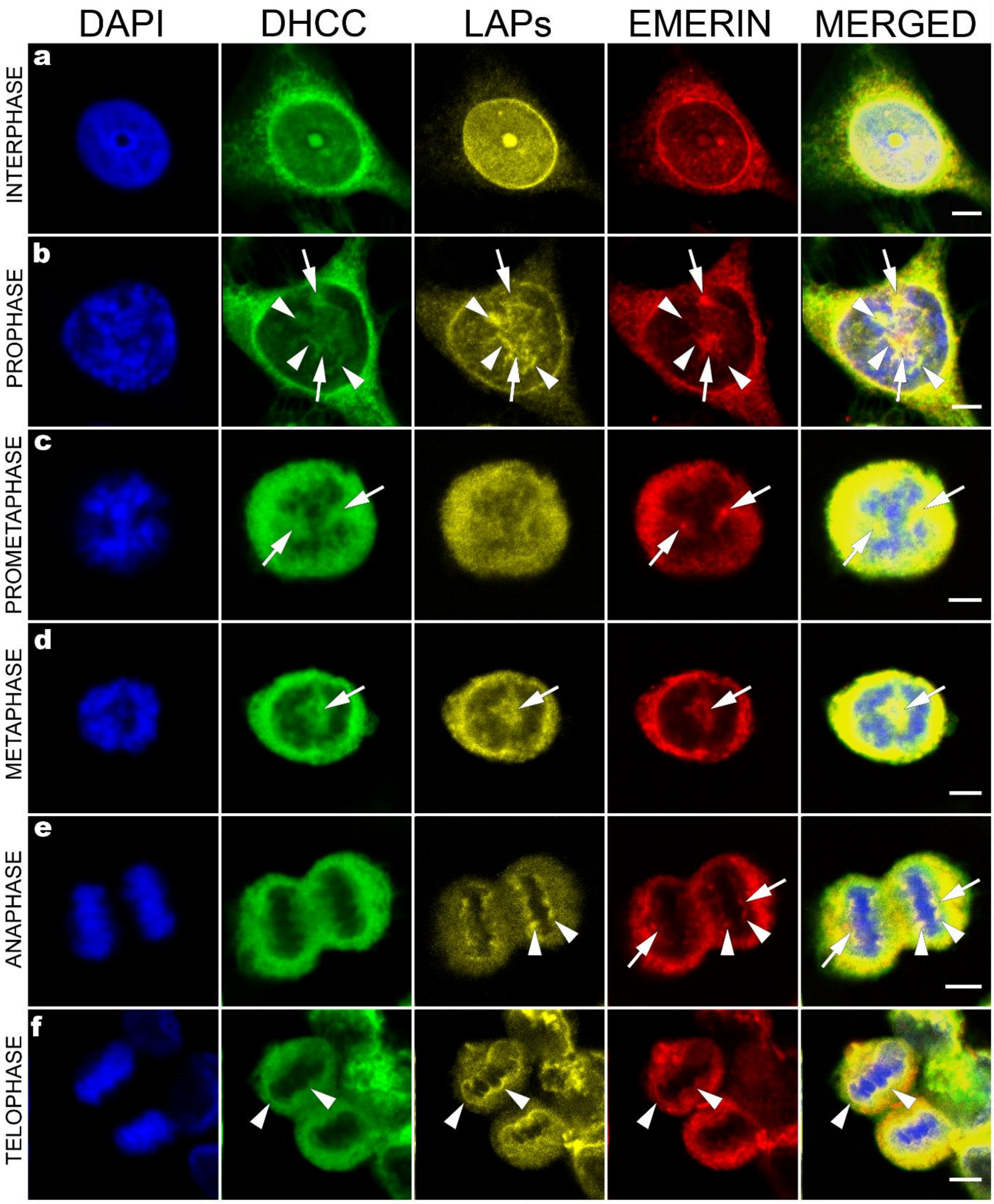

Figure S2. Cell cycle–dependent distribution of membranes, LAP2, and emerin in HeLa cells. Although LAP2β is an integral membrane protein and interacts with lamins and BAF (and emerin), its distribution during prophase, prometaphase, and metaphase is different. LAP2β follows in general the distribution of membranes, and also is associated with mitotic spindle space (prophase, prometaphase). The emerin subfraction associates more strongly with the mitotic spindle space (centrosomes and tubulin) than with membranes. Arrowheads point specific location for fractions of emerin, LAP2β and membranes in mitosis. Arrows show the centrosomal areas. For more details see main text. HeLa cells were grown on coverslips, fixed with 4% PFA, and stained for emerin, LAPs, and membranes (DHCC). DNA was visualized with DAPI (blue). Single confocal sections (1.5 μm) through the center of cells are shown. Bar, 5 μm.

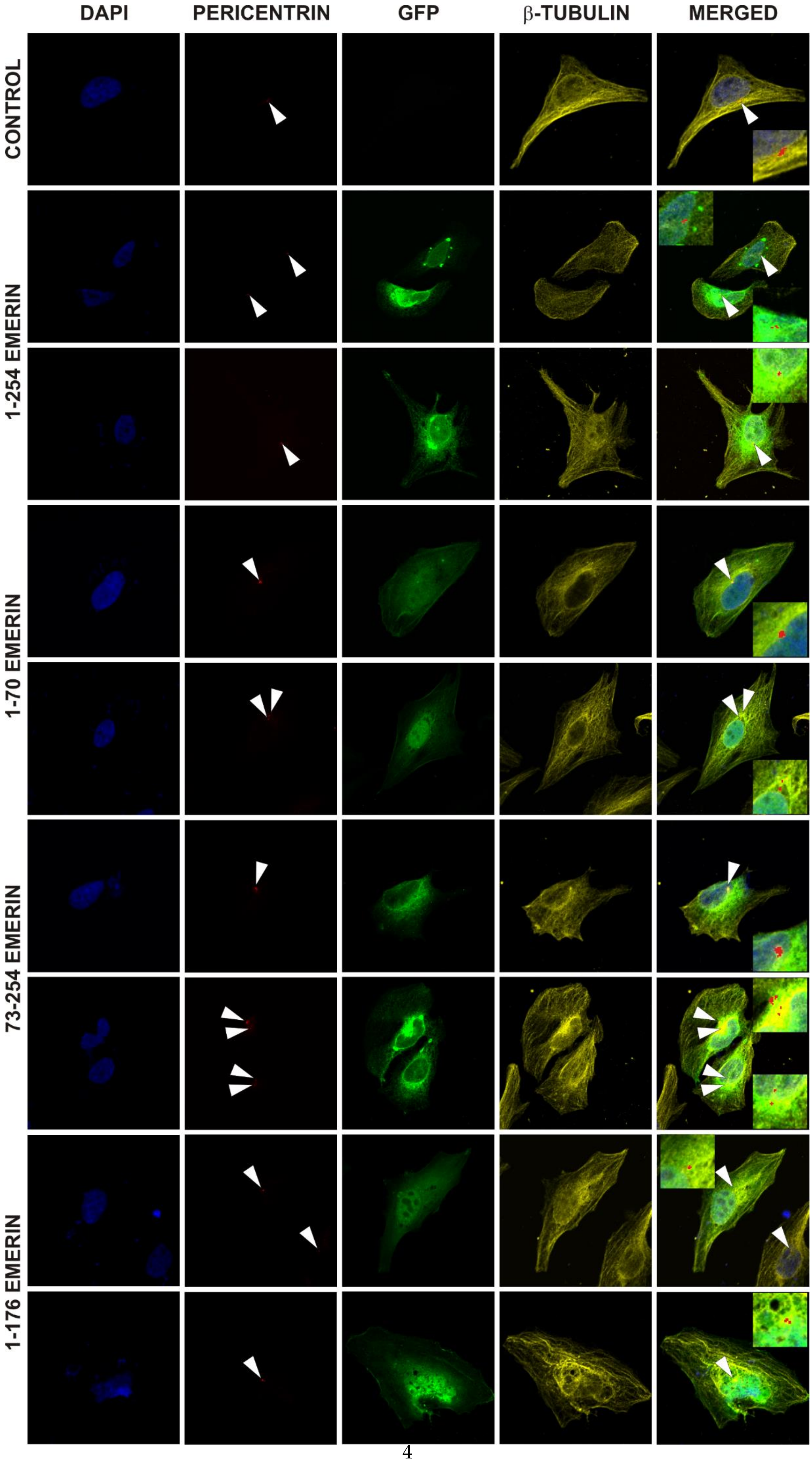
DAPI
PERICENTRIN
GFP
β-TUBULIN
MERGED
CONTROL
1-254 EMERIN
1-70 EMERIN
73-254 EMERIN
1-176 EMERIN

Figure S3. Transient transfection of HeLa cells with emerin deletion mutant constructs affects the location of centrosomes and tubulin network. Typically, the, most frequent phenotypes of cells transfected with EGFP-emerin deletion mutants were selected in order to demonstrate the fate and distribution of centrosomes and the tubulin network in transfected cells. Insets present magnification of centrosome region. The pericentrin signal was enhanced by adding a layer containing corresponding red dots and pointed with arrowheads additionally. HeLa cells were transfected with plasmids coding for EeGFP fusion proteins (pEGFP-E1-70, pEGFP-E1-176, pEGFP-E73-254, or pEGFP-E1-254). After 48 h, cells were stained for pericentrin, marking the location of centrosomes (red) and tubulin (yellow). EGFP fluorescence of the fusion protein was used to detect the location of transfected protein (green). Nuclei were stained for DNA with DAPI (blue). Insert in the merged image demonstrates at magnification the location and distribution of pericentrin (red). Phenotypes were documented using laser confocal microscopy. Sections (1.5 μm) through the nuclei, but centered at pericentrin staining, were selected for documentation.

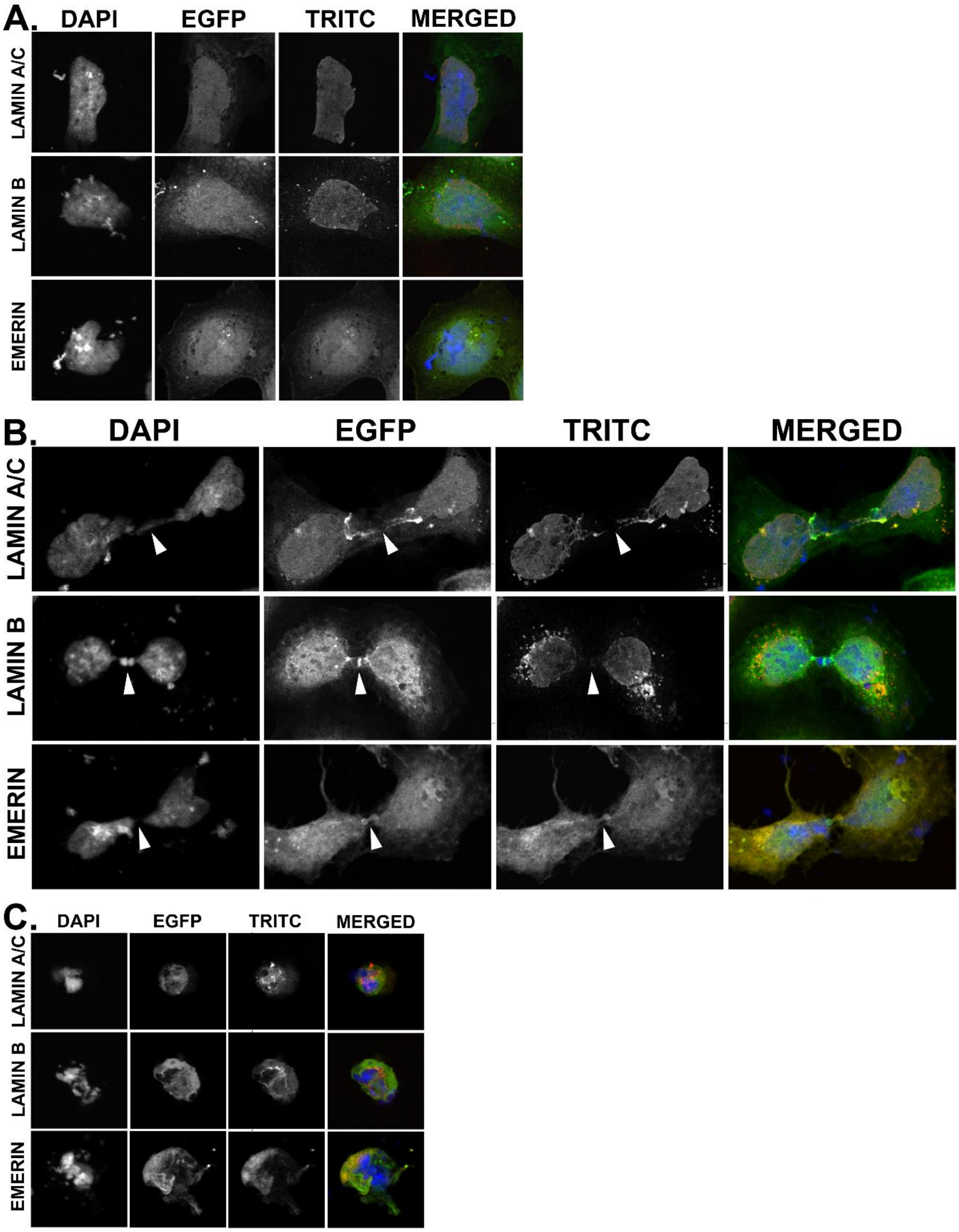

Figure S4. Demonstration of typical phenotypes classified as "abnormal", "cut" and "apoptotic" induced by expression of emerin deletion mutants using E70 mutant as an example. Emerin E70 deletion mutant localized to nucleoplasm and cytoplasm and induced changes in nuclear shape and redistribution of endogenous lamins A and B. Only typical phenotypes illustrating examples of particularly defined phenotypes induced by E70 transfection arehave been shown. HeLa cells were transiently transfected with EGFP-emerin deletion mutant E70 and stained 48 h after post-transfection for endogenous lamin A/C, lamin B1, and emerin (protein staining by antibody was visualized in red –– TRITC). The transfected fusion protein was visualized by EGFP fluorescence (green). Nuclei/DNA was counterstained with DAPI (blue). Atypical phenotypes were documented using laser confocal microscopy. Middle sections (1.5 μm) through the center of the nuclei were

selected for documentation. Bar, 5 μm. A, "Abnormal" shaped cells; B, "cut" phenotype; and C, apoptotic cells. Arrowheads indicate not properly separated chromatin in midbody area for "cut" phenotype cells.

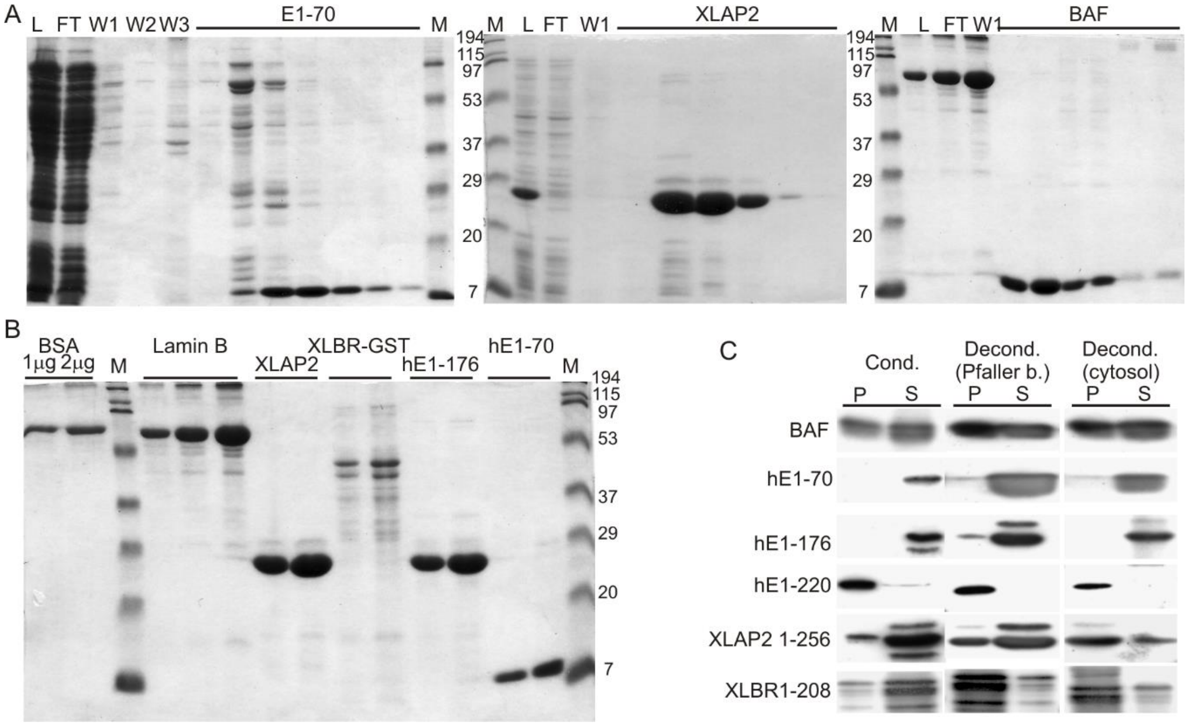

Figure S5. *Xenopus* sperm chromatin *in vitro* binding assay of chromatin association with emerin deletion mutants and other well-known chromatin-binding nuclear lamina proteins. A. Typical preparation and isolation of bacterially expressed proteins using His-tag affinity chromatography followed by resolution of proteins by SDS PAGE and Coomassie staining. L – load; FT – flow through; W – wash. B. Quality of bacterially expressed proteins used in the assay together with BSA loading control. Proteins were resolved by SDS PAGE followed by Coomassie staining. C. Demembranated sperm chromatin was used for analyses of chromatin binding efficiency of bacterially expressed emerin deletion mutants and control nuclear lamina proteins. Three types of chromatin were used: plain, demembranated, condensed chromatin; demembranated chromatin decondensed with Pfaller buffer containing poly-glutamic acid; and decondensed chromatin using cytoplasm from *Xenopus* egg extract enriched with ATP generating system. After incubation with bacterially expressed protein, chromatin was pelleted by centrifugation and both pellet (P) and supernatant (S) were analyzed for associated proteins using western blotting with antibodies for BAF, emerin, XLAP2, and XLBR.
